# A Reactive and Specific Sensor for Activity-Based ^19^F-MRI Sensing of Zn^2+^

**DOI:** 10.1021/acssensors.4c01895

**Published:** 2024-10-24

**Authors:** Lucia M. Lee, Nishanth D. Tirukoti, Balamurugan Subramani, Elad Goren, Yael Diskin-Posner, Hyla Allouche-Arnon, Amnon Bar-Shir

**Affiliations:** † Department of Molecular Chemistry and Materials Science, Weizmann Institute of Science, Rehovot 7610001, Israel; ‡ Department of Chemistry, Queen’s University, Kingston, Ontario K7L 3N6, Canada; § Calico Life Sciences LLC, 1170 Veterans Boulevard, South San Francisco, California 94080, United States; ∥ Department of Chemical Research Support, Weizmann Institute of Science, Rehovot 7610001, Israel

**Keywords:** ^19^F-MRI, Zn^2+^ imaging, Activity Based Sensing, Responsive Agents, Metal Ion Sensing

## Abstract

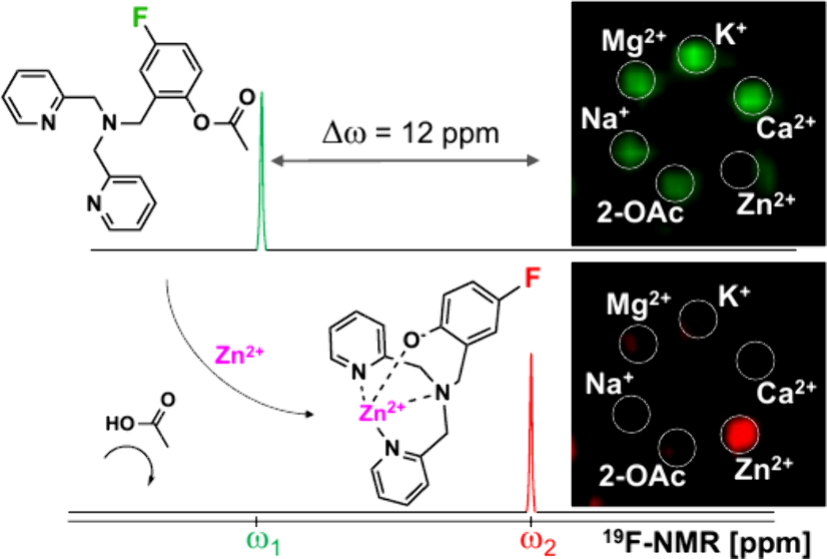

The rapid fluctuations of metal ion levels in biological systems are faster than the time needed to map fluorinated sensors designed for the ^19^F-MRI of cations. An attractive modular solution might come from the activity-based sensing approach. Here, we propose a highly reactive but still ultimately specific synthetic fluorinated sensor for ^19^F-MRI mapping of labile Zn^2+^. The sensor comprises a dipicolylamine scaffold for Zn^2+^ recognition conjugated to a fluorophenyl acetate entity. Upon binding to Zn^2+^, the synthetic sensor is readily hydrolyzed, and the frequency of its ^19^F-functional group in ^19^F-NMR is shifted by 12 ppm, allowing the display of the Zn^2+^ distribution as an artificial MRI-colored map highlighting its specificity compared to other metal ions. The irreversible Zn^2+^-induced hydrolysis results in a “turn-on” ^19^F-MRI, potentially detecting the cation even upon a transient elevation of its levels. We envision that additional metal-ion sensors can be developed based on the principles demonstrated in this work, expanding the molecular toolbox currently used for ^19^F-MRI.

The vast majority of imaging probes developed for metal ion sensing comprise two entities: a multidentate organic ligand for cation recognition and binding, and an imageable entity that generates a readable signal.^1,2^ Adopting this design principle, responsive paramagnetic contrast agents were developed to monitor labile cations with magnetic resonance imaging (MRI),^3−8^ overcoming the restrictions of fluorescent imaging and enabling *in vivo* investigation of metal ions in deep tissues. In these agents, the ion recognition entity, which participates in the paramagnetic metal coordination, binds the cation of interest, freeing the paramagnetic center to bind water molecules, thus inducing paramagnetic relaxation enhancement and a change in the MRI contrast.^9^ Optimization of the structural and binding properties of such MRI-responsive agents allowed spatial mapping of dynamic changes in Ca^2+^ levels in the brain,^10−13^ Cu^2+^ in the liver,^14^ and Zn^2+^ in pancreatic^15−17^ and prostate^18,19^ tissues *in vivo*. While these designs benefit from high sensitivity, relying on two different molecular entities, one for cation binding and one for MRI signal amplification, limits the flexibility of the sensor architecture. In addition, the large ^1^H-MRI signal of the surrounding tissue may complicate the interpretation of results and their quantification when using paramagnetic contrast agents.

Fluorinated ligands were proposed as an alternative type of cation sensor where the ion-binding entity also serves the signal generator, aiming to overcome some of these limitations.^20^ With this type of sensor, upon binding the cation of interest, a significant chemical shift offset in the ^19^F-NMR spectrum is obtained with a unique MR fingerprint for each ion, allowing the monitoring of multiple ions simultaneously with the aid of a single sensor. Extending this approach for ^19^F-MRI studies, combined with the chemical exchange saturation transfer (CEST) contrast mechanism^21,22^ has shown promise for noninvasive *in vivo* mapping of labile Zn^2+^ in prostate^23^ and brain^24^ tissues. Although fluorinated agents provide background-free maps and quantifiable information, which do not apply to paramagnetic MRI-responsive agents, the time needed to acquire their signal is expected to be longer than the changes in metal ion levels in biological systems, thus calling for advances for this group of sensors.

An emerging concept for sensing, which relies on the reactivity of the sensor rather than on molecular recognition and reversible binding, may overcome some of the remaining challenges for the ^19^F-MRI of metal ions. In this approach, activity-based sensing (ABS),^25^ the analyte identification occurs via an analyte-initiated reaction, after which a detectable signal is generated.^26−29^ Applying the ABS principles to ^19^F-MRI has shown potential for mapping the activity of several key enzymes^30−32^ and biologically relevant redox conditions.^33,34^ The irreversible rapid change in the ^19^F-MR properties of the sensor upon conversion creates “turn-on” MRI signals that can be repeatedly acquired, thus overcoming the relatively low sensitivity of the ^19^F-MRI approach, which frequently requires long acquisition times. Recognizing the need for advanced and specific MRI-responsive agents for metal ions with biological relevance, we propose a highly reactive but still very specific sensor for the ^19^F-MRI mapping of labile Zn^2+^. Upon binding to Zn^2+^, the synthetic sensor is readily hydrolyzed, and the resonance of its ^19^F-functional group is shifted by 12 ppm, allowing the display of the Zn^2+^ distribution as an artificial MRI-colored map highlighting its ultimate specificity.

Aiming to obtain a sensor with specific ^19^F-NMR properties that shows a different ^19^F-NMR chemical shift (Δω) upon Zn^2+^-catalyzed hydrolysis, a fluorinated molecular probe was proposed (Figure 1a,b). To this end, a dipicolylamine (DPA) scaffold was used as a zinc-recognition entity conjugated to a fluorinated phenyl acetate moiety. Based on previous designs, we hypothesized that upon binding to a DPA entity,^26^ the intramolecular interactions between the bound Zn^2+^ and the acetate’s carbonyl group, along with the Lewis acidity of the ion, would catalyze the hydrolysis of the ester. This hydrolysis is expected to result in a much more stable phenolic complex of Zn^2+^ and induce a change in the ^19^F-NMR chemical shift of the probe. Two sensors were obtained, each with an acetate functional group at the phenyl ring and a fluorine substitution at either the *meta*-position (**1-OAc**) or the *para*-position (**2-OAc**), relative to the acetate group. Then, aqueous solutions of the two sensors were subjected to ^19^F-NMR measurements without or with the addition of Zn^2+^. Upon Zn^2+^-induced hydrolysis of the acetate group in **1-OAc**, only a small change in the ^19^F-NMR chemical shift (Δω = 0.3 ppm) was observed from the resulting **1-OH-Zn**^**2+**^ complex (Figure 1c). In contrast, a significantly large Δω of 12 ppm was detected in the ^19^F-NMR spectrum after the hydrolysis of **2-OAc** to **2-OH** in the presence of Zn^2+^ (Figure 1d). This large Δω, which was applicable for ^19^F-MR sensing of Zn^2+^ based on reversible binding,^35^ allows a clear separation between the two forms of the sensor before and after Zn^2+^-induced hydrolysis.

**Figure 1:**
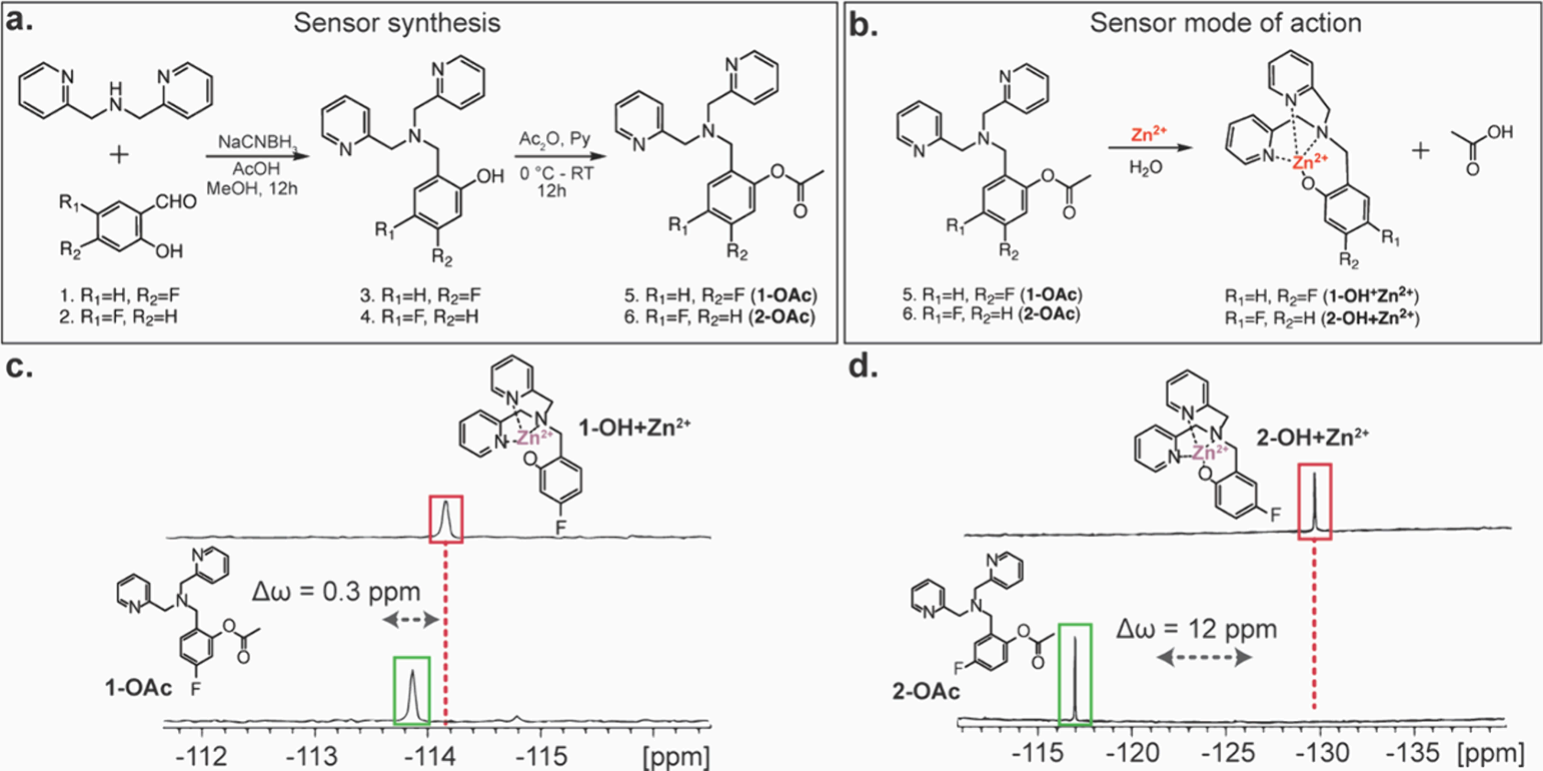
Zn^2+^ sensor synthesis and mode of action. (a) The synthetic route for synthesizing 1-OAc and 2-OAc. (b) Illustration of the sensor hydrolysis and its mode of action. (c) ^19^F-NMR spectra of 1-OAc (labeled green) and the obtained 1-OH-Zn^2+^ complex (labeled red) upon hydrolysis, with the chemical shift offset between the two forms, **Δω** = 0.3 ppm(d) ^19^F-NMR spectra of 2-OAc (labeled green) and the obtained 2-OH-Zn^2+^ complex (labeled red), **Δω** = 12 ppm.

The white-transparent crystals of the Zn^2+^-complexes were obtained by crystallizing either **1-OH** or **2-OH** in the presence of Zn(ClO_4_)·6H_2_O in methanol at ambient temperature, and the solid-state structures of the complexes of **1-OH** and **2-OH** with Zn^2+^ were obtained and studied (Figures 2, S1, and S2 and Table S1).

**Figure 2:**
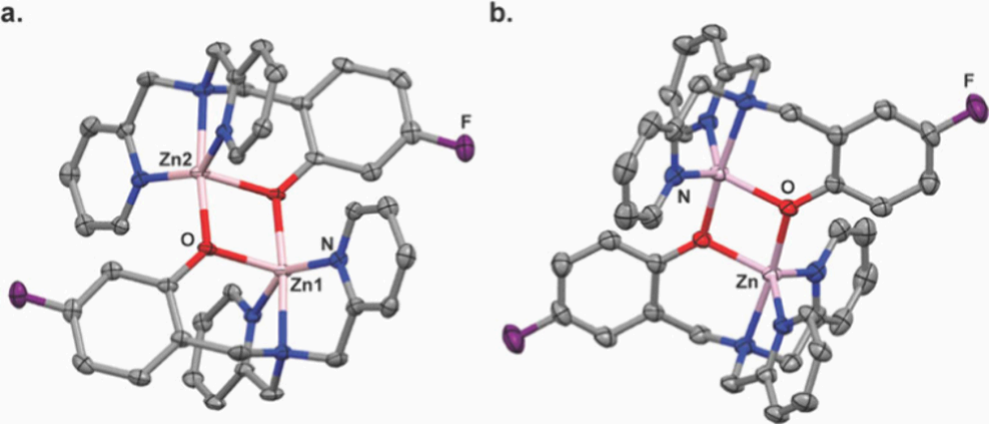
Crystal structures of complexes of Zn^2+^ with 1-OH and 2-OH. The ORTEP schemes for the dimers obtained are shown in (a) 1-OH-Zn^2+^ and (b) 2-OH-Zn^2+^. Displacement ellipsoids are shown at the 50% probability level, and hydrogen atoms have been omitted for the sake of clarity. Color codes: F^**–**^ (purple), O (red), N (blue), and Zn^2+^ (pink).

X-ray crystallography revealed a dimeric structure for both the **1-OH-Zn**^**2+**^ and **2-OH-Zn**^**2+**^ complexes. In **2-OH-Zn**^**2+**^, the preferable molecule for ^19^F-MRI (Figure 1d), the Zn^2+^ center is coordinated to three nitrogen atoms from the dicopylamine and two bridging oxygen atoms of fluoro-phenols. The Zn–N bond lengths range between 2.054(2) Å, 2.056(2) Å, and 2.165(2) Å, while the Zn–O bond lengths are 1.995(2) Å, comparable to those found in a previously reported Zn^2+^ fluorescent sensor,^36^ demonstrating the strong coordination ability of **2-OH’s** to the Lewis acidic Zn^2+^ ions in **2-OH-Zn**^**2+**^.

The specificity of the **2-OAc** for Zn^2+^ sensing was examined with potentially competitive cations (Figure S3), and the ^19^F-NMR spectra of the **2-OAc** solution in the presence of Ca^2+^, Mg^2+^, Cu^2+^, Fe^2+^, Fe^3+^, Mn^2+^, Ni^2+^, Co^2+^, Na^+^, or K^+^ were compared to its spectrum in the presence of Zn^2+^. Notably, only the Zn^2+^-containing solution yielded a characteristic ^19^F-NMR peak at −129 ppm, which is assigned to the **2-OH-Zn**^**2+**^ complex. No significant ^19^F-NMR peak could be assigned to a **2-OH-M**^**+**^ complex in the solution for all of the other studied cations. For the paramagnetic cations examined, either a small shift or a line-broadening could be detected for the ^19^F-NMR peak of the fluorinated sensor.

Then, the kinetic properties of the **2-OAc** hydrolysis in the presence of Zn^2+^ were compared to that of Ca^2+^, Cu^2+,^ and Fe^2+^ (Figure 3 and Figure S4). To this end, buffered (HEPES buffer, pH 7.2) solutions of 3 mM **2-OAc** in equimolar cation concentrations were prepared, and consecutive ^19^F-NMR spectra were measured. Clearly, 10 min after the Zn^2+^ addition, when the acquisition of the first ^19^F-NMR was completed, a peak at −129 ppm, a characteristic resonance of the **2-OH-Zn**^**2+**^ complex, was already obtained (Figure 3a and Figure S4a). Over time, the intensity of the ^19^F-NMR peak of **2-OAc** (−117 ppm) was reduced until its elimination, while the intensity peak of the **2-OH-Zn**^**2+**^ complex was elevated as expected from continuous hydrolysis of **2-OAc** by the labile-free Zn^2+^ in the solution. This observation was not detected in the presence of the other cations studied (Figure 3b–d, Figure S4b–d). Even for ions expecting to bind a dipicolylamine scaffold, such as Fe^2+^ and Cu^2+^,^37^ although some paramagnetic line-broadening and reduction in the intensity of the **2-OAc** peak were obtained over time, no evidence of **2-OH** complexes in the presence of these cations was shown by ^19^F-NMR (Figure 3c,d and Figure S4). Still, traces of such complexes were identified by mass spectroscopy measurements (Figures S5–S7). Importantly, even at a much lower Zn^2+^ concentration, a pronounced peak at the resonance of the **2-OH-Zn**^**2+**^ complex (−129 ppm) could be detected in addition to the nonhydrolyzed **2-OAc** compound with its characteristic peak at −117 ppm (Figure S8). Notably, in this case, when the concentration of Zn^2+^ was lower than that of the **2-OAc** compound, an additional peak of the hydrolyzed **2-OH** compound without Zn^2+^ bound to it was detected at −126 ppm. This observation could be explained by the reversible binding of Zn^2+^ to **2-OH**.

**Figure 3:**
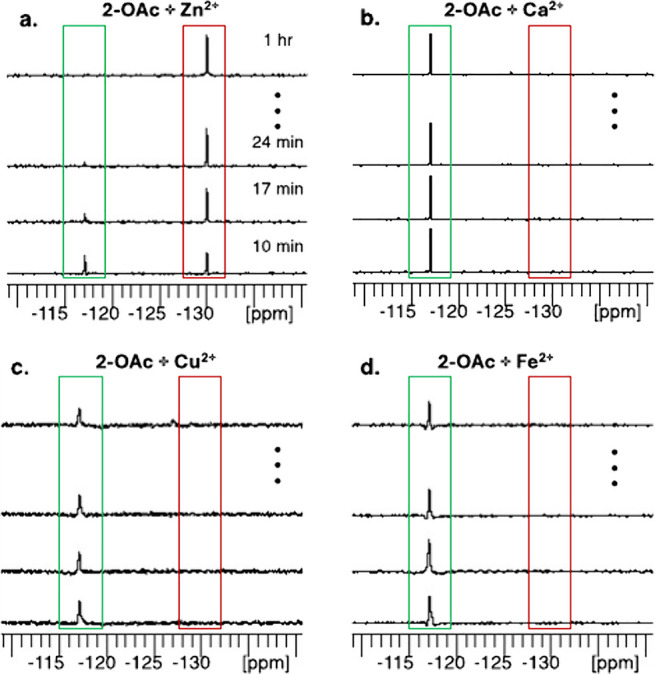
Fluorinated sensor specificity. Real-time ^19^F NMR spectra of 2-OAc in the presence of (a) Zn^2+^, (b) Ca^2+^, (c) Cu^2+^, and (d) Fe^2+^. The green rectangle marks the chemical shift of 2-OAc (−117 ppm), and the red rectangle marks the chemical shift of 2-OH-Zn^2+^ (−129 ppm).

The kinetic profile of Zn^2+^-induced hydrolysis of **2-OAc** was studied by ^19^F-NMR (Figure 4). The half lifetime (*t*_1/2_) of **2-OAc** in the presence of Zn^2+^ was then evaluated by plotting the integrals of the two obtaining peaks at the ^19^F-NMR spectra, −117 ppm before Zn^2+^-induced hydrolysis and −129 ppm after the hydrolysis. The *t*_1/2_ of **2-OAc** in the presence of Zn^2+^ at 25 °C was 9 min (Figure 4a,d) while that at 37 °C was 4 min (Figure 4b,d and Figure S9). Importantly, with the addition of 3 mM Ca^2+^, the *t*_1/2_ of **2-OAc** was found to be longer than 30 h, reflecting the sensor stability in an aqueous solution and its specificity for Zn^2+^ (Figure 4c and Figure S10). At acidic conditions (pH = 6.5), although slower than at physiological pH, the *t*_1/2_ of **2-OAc** in the presence of Zn^2+^ (at 37 °C) was still relatively short (*t*_1/2_ = 9 min, Figure S11). As expected, the Zn^2+^-induced hydrolysis was faster in alkaline conditions at physiological temperature, with a 100% conversion of **2-OAc** to **2-OH** before the acquisition of the first ^19^F-NMR spectrum was completed (Figure S11). Prominently, under strong basic conditions (pH = 8.5), no hydrolysis of **2-OAc** occurred without Zn^2+^, showing the cruciality of the ion in the reaction and the stability of **2-OAc** even at an elevated pH (Figure S12).

**Figure 4:**
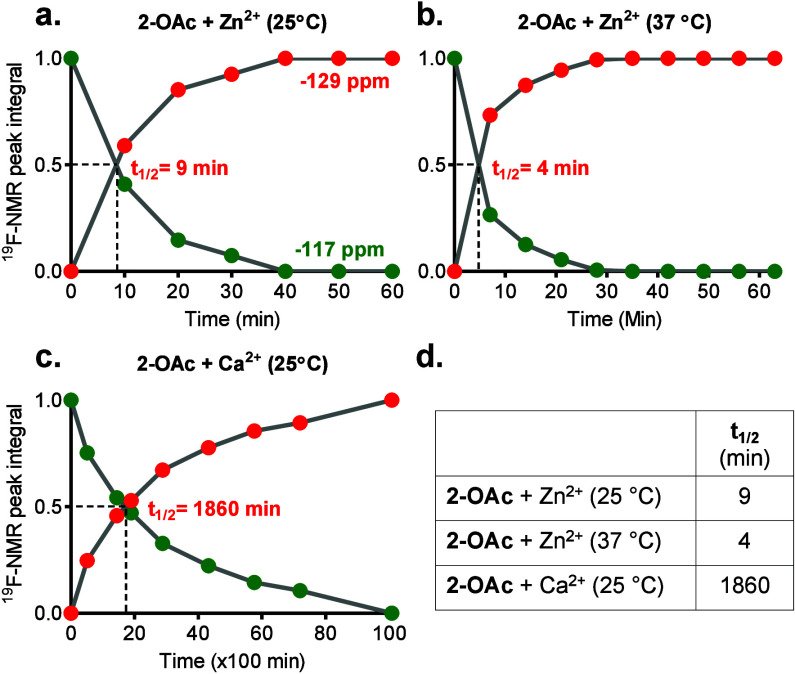
Kinetic studies of the 2-OAc activity. ^19^F-NMR signal intensity representing the conversion of 2-OAc (green dots, ^19^F-NMR peak at −117 ppm) to 2-OH (red dots, ^19^F-NMR peak at −129 ppm) in the presence of Zn^2+^ at (a) 25 or (b) 37 °C; or in the presence of (c) Ca^2+^ at 25 °C. (d) The *t*_1/2_ values of 2-OAc as evaluated from the plots in panels a–c.

Similar observations were obtained when the same assay was performed in a cell culture medium (DMEM) containing physiologically relevant concentrations of nucleophilic metabolites (sugars, amino acids, and salts) and 10% fetal bovine serum albumin (FBS). Remarkably, when such a medium contained Zn^2+^, **2-OAc** was hydrolyzed entirely before the first ^19^F-NMR spectrum was acquired (Figure S13). This contrasts with the exact solution of **2-OAc**, to which Zn^2+^ was not added, where even at 37 °C, only a slight hydrolysis of **2-OAc** could be detected after 1 h (Figure S14). This observation that serum albumin content accelerates the Zn^2+^-induced hydrolysis of **2-OAc**, should be further studied. Nevertheless, it could be attributed to the serum albumin’s ability to stabilize Zn^2+^-bound DPA scaffolds, as previously reported.^4^ This stabilization might strengthen the intramolecular interactions between the bound Zn^2+^ and the acetate’s carbonyl group, thereby accelerating the ester hydrolysis due to the proximity of the Lewis acid (bound Zn^2+^) in the formed **2-OAc-Zn**^**2+**^ complex. Overall, these results show the stability of **2-OAc** in the absence of Zn^2+^, regardless of the solution conditions with no apparent hydrolysis without the ion at elevated temperature, alkaline conditions, and the presence of high concentration of nucleophiles and serum protein assuring that the proposed sensor will be stable in a biological environment.

Finally, we set out to examine the ability to map the presence of **2-OAc** with ^19^F-MRI and present its Zn^2+^-sensing capabilities in a multiplex manner based on the large chemical shifts of the sensor before and after its hydrolysis. For that purpose, a phantom of six tubes containing 3 mM **2-OAc** and an equimolar of a different cation (one tube was used as a negative control without cation addition) was set and studied. Figure 5a shows the ^19^F-NMR spectra of five tubes containing **2-OAc** and a cation in HEPES buffered solution (pH = 7.2). As clearly shown, only for the solution that contained Zn^2+^ a ^19^F-NMR peak was observed at −129 ppm, as expected from a **2-OH-Zn**^**2+**^ complex, reflecting the “turn-on” ^19^F-MR feature of the Zn^2+^ sensor. A single and clear peak is obtained for all other tubes with a resonance of −117 ppm, reflecting intact, nonhydrolyzed **2-OAc** in the studied solution. ^1^H-MRI of the studied phantom showed no difference between the tubes (Figure 5b).

**Figure 5:**
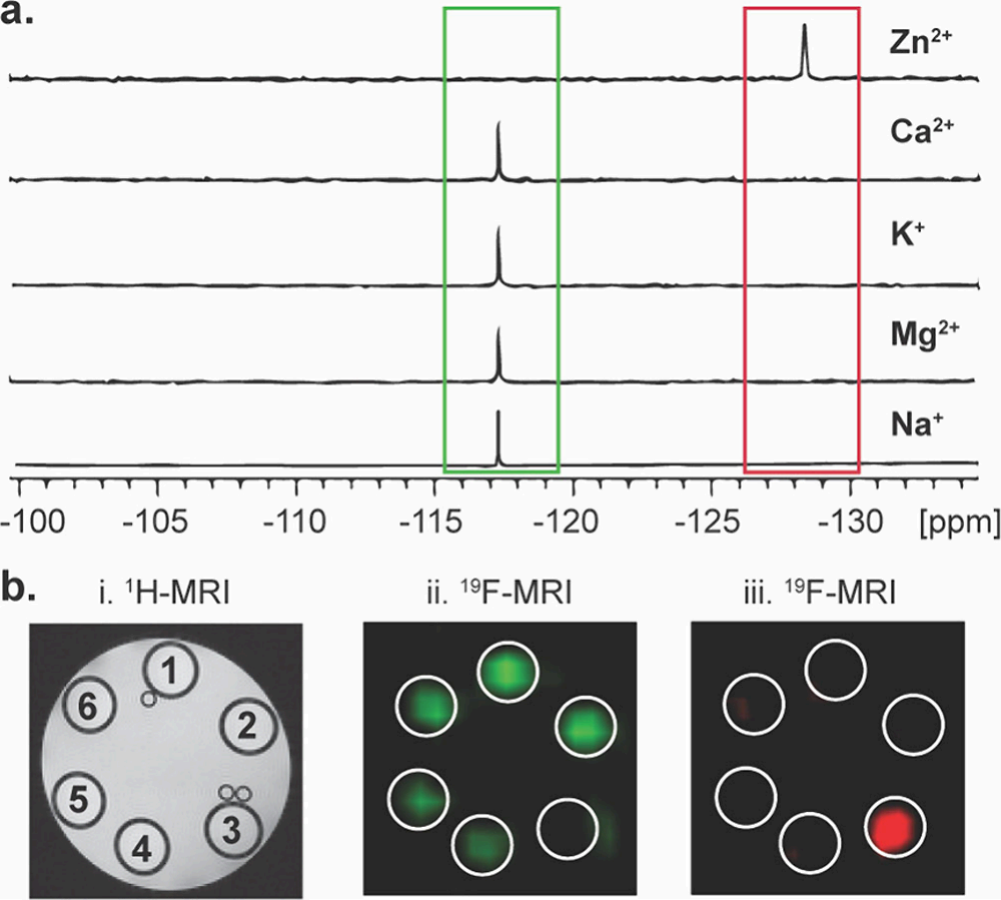
Multiplexed ^19^F-MRI of Zn^2+^ sensing. (a) ^19^F NMR spectra of 3 mM 2-OAc in the presence of 3 mM Zn^2+^, Ca^2+^, K^+^, Mg^2+^ and Na^+^ at 37 °C. (b) ^1^H-MRI (left) of the studied phantom composed of six tubes containing 3 mM 2-OAc and 3 mM cation, i.e., K^+^ (#1), Ca^2+^(#2), Zn^2+^(#3), Na^+^ (#5), and Mg^2+^ (#6). Tube #4 contained only 2-OAc. ^19^F-MRI map as obtained with the O_1_ set to −117 ppm (middle panel, green) and or −129 ppm (right panel, red).

When ^19^F-MRI data was acquired with the center frequency (O_1_) set to the resonance of **2-OAc**, i.e., 117 ppm, a clear signal was obtained from 5 out of the 6 studied tubes. In contrast, setting up the O_1_ of the ^19^F-MRI acquisition protocol to 129 ppm, a ^19^F-MR signal was obtained only from the tube containing Zn^2+^, a clear indication of the complex of the cation with **2-OH**. In contrast to the ^19^F-CEST approach, which requires the acquisition of at least two ^19^F-MR images for Zn^2+^ mapping (“on-resonance” and “off-resonance”),^24^ the approach presented here can provide ^19^F-MR map of Zn^2+^ distribution by setting up the O_1_ of the ^19^F-MRI acquisition protocol to −129 ppm and acquiring a single ^19^F-MR image.

In addition to this advantage, the large Δω of 12 ppm between in the ^19^F-MR resonances of nonhydrolyzed **2-OAc** and the Zn^2+^ complex of the sensor after its hydrolysis to **2-OH** allowed us to present the results in artificial MRI colors, with green representing the ^19^F-MRI map at −117 ppm and red representing the ^19^F-MRI map at −129 ppm. This frequency encoding feature resulting in multicolor representations of different species is unique to ^19^F-MRI studies and is useful for mapping multiple targets in the same region of interest,^38^ but it can also be used for other applications.^39−41^ To summarize this part, we showed that **2-OAc** could be used as a sensor for activity-based ^19^F-MRI mapping of Zn^2+^ with the capability to present the existence of the cation in artificially colored MRI maps capitalizing on the distinctive chemical shift of the resulted **2-OH-Zn**^**2+**^ complex.

In conclusion, we demonstrated a conceptually novel approach for MRI sensing of labile Zn^2+^, which relies on ABS principles. Specifically, having designed a fluorine-modified phenyl acetate moiety attached to a dipicolylamine motif, a Zn^2+^ sensitive ^19^F-MRI sensor (**2-OAc**) was obtained. We showed that upon Zn^2+^ recognition and binding, **2-OAc** readily undergoes hydrolysis to result in the **2-OH-Zn**^**2+**^ complex. The large ^19^F-NMR Δω difference between the resonances of **2-OAc** and that of **2-OH-Zn**^**2+**^ allows one to spectrally resolve them toward their presentation in a dual-color ^19^F-MRI fashion, obtaining a “turn-on” ^19^F-MRI sensor for Zn^2+^. The high specificity and reactivity of **2-OAc** only in the presence of Zn^2+^ to obtain, nonreversibly, **2-OH** makes the proposed strategy advantageous for ^19^F-MRI, which frequently requires acquisition times much longer than the time dynamic biological processes occur. We envision that the principles shown in this work for ABS ^19^F-MRI sensing of Zn^2+^ could be generalized for imaging other metal ions.

## Data Availability

CCDC 2366644–2366645 contains the supplementary crystallographic data for this paper. These data can be obtained free of charge via www.ccdc.cam.ac.uk/data_requests/cif, by emailing data_request@ccdc.cam.ac.uk, or by contacting The Cambridge Crystallographic Data Centre, 12 Union Road, Cambridge CB2 1EZ, UK; fax: + 44 1223 336033.

## Supporting Information Available

Sensors synthesis and characterizations are provided, along with additional experimental methods and supporting figures (PDF)
