# Supplementary material for: A Reactive and Specific Sensor for Activity-Based 19F-MRI Sensing of Zn2+
Source: ACS Sens. 2024 Oct 24;9(11):5770–5. doi: 10.1021/acssensors.4c01895 (PMC11590105; doi:10.1021/acssensors.4c01895)
Supplement: Supplementary file 1 — se4c01895_si_001.pdf [file se4c01895_si_001.pdf]

Supporting Information

A Reactive and Specific Sensor for Activity-Based ^{19}F-MRI Sensing of Zn^{2+}

Lucia M. Lee[1,2], Nishanth D. Tirukoti[1,3], Balamurugan Subramani[1], Elad Goren[1], Yael Diskin-Posner[4], Hyla Allouche-Arnon[1], and Amnon Bar-Shir[1*]
[1]Department of Molecular Chemistry and Materials Science, Weizmann Institute of Science, Rehovot, 7610001, Israel
[2]Current address: Department of Chemistry, Queen's University, Kingston, K7L 3N6, Canada.
[3]Current address: Calico Life Sciences LLC, 1170 Veterans Boulevard, South San Francisco, California, 94080, U. S. A.
[4]Department of Chemical Research Support, Weizmann Institute of Science, Rehovot, 7610001, Israel.
[*]Corresponding Author: amnon.barshir@weizmann.ac.il

Table of Contents

A. **Materials and Methods**....2
B. **Chemical Synthesis Procedure**....2
Compound 2-OH....2
Compound 1-OH....2
Compound 2-OAc....3
Compound 1-OAc....3
C. **NMR and MRI Experiments**....3
D. **X-ray crystallography**....4
Compound 2-OAc + Zn^{2+} Complex....4
Compound 1-OAc + Zn^{2+} Complex....4
E. **Supporting Tables**....5
Table S1....5
Table S2....6
F. **Supporting Figures (S1-S32)**....7
Figure S1....7
Figure S2....8
Figure S3....9
Figure S4....10
Figure S5....11
Figure S6....11
Figure S7....12
Figure S8....12
Figure S9....13
Figure S10....13
Figure S11....14
Figure S12....14
Figure S13....15
Figure S14....15
Figure S15....16
Figure S16....16
Figure S17....16
Figure S18....17
Figure S19....18
Figure S20....18
Figure S21....18

Figure S22 19
Figure S23 20
Figure S24 20
Figure S25 20
Figure S26 21
Figure S27 21
Figure S28 22
Figure S29 22
Figure S30 22
Figure S31 23
Figure S32 23
G. **Supplementary References** 24

A. Materials and Methods.

All reagents and solvents were received from commercial suppliers. 5-fluoro-2-hydroxybenzaldehyde, 2-fluoro-6-hydroxybenzaldehyde and 2,2'-dipicolylamine were purchased from Tzamal D-Chem Laboratories Ltd. Sodium cyanoborohydride was purchased from Sigma Aldrich. Deuterated solvents ($CDCl_3$, D_2O) were purchased from Cambridge Isotope Laboratories Inc. (Andover, MA). The ^1H, ^{13}C and ^{19}F NMR spectra, ^{19}F-NMR Zn^{2+} binding studies were recorded on Bruker 9.4 T 0r 11.7 T NMR spectrometers (as specifically noted). High-resolution mass spectrometry (HR-MS) was recorded on an AB SCIZ 5800 MALDI TOF instrument at the Weizmann Institute of Science mass spectrometry facility.

B. Chemical Synthesis Procedure.

Compound 2-OH: Solutions of 2-2'-dipicolylamine (1.2g, 6.02 mmols) and 5-fluoro-2-hydroxybenzaldehyde (0.84g, 6.02 mmols) in methanol were degassed with nitrogen before mixing. A few drops of glacial acetic acid and sodium cyanoborohydride (0.19g, 3.01 mmols) were then added to the mixture, which was stirred at 70°C for 72 h. After confirming the completion of the reaction by thin-layer chromatography (TLC), the solvent was evaporated under reduced pressure. The crude product was dissolved in chloroform and extracted with a saturated solution of Na_2CO_3 with $CHCl_3$ (3x50 mL), then the combined organic phase was dried over Na_2SO_4 and filtered. The concentrated organic phase was purified using silica gel column chromatography (EtOAc/Ether, 30:70) to obtain compound **2-OH** as an orange oil (1.61g, 83%). ^1H NMR (400.35 MHz, $CDCl_3$): 8.58 (m, 2H), 7.64 (m, 2H), 7.36 (m, 3H), 7.18 (m, 2H), 7.08 (m, 1H), 6.93 (m, 1H), 6.79 (m, 1H), 3.90 (s, 4H) and 3.81 (s, 2H). $^{13}C\{^1H\}$ and DEPTQ$\{^1H\}$ NMR (100.67 MHz, $CDCl_3$): 157.99, 156.98, 154.64, 153.57, 153.55, 148.89, 136.89, 122.82, 123.76, 123.25, 122.33, 117.24,117.16, 116.49,116.26, 115.30, 115.08, 58.96, 56.50. ^{19}F (376.66 MHz, $CDCl_3$): -127.42. HRMS (ESI) (m/z): $[M+H]^+$ calcd. For $C_{19}H_{19}N_3OF$ 324.1512, found 324.1501.

Compound 1-OH: A similar procedure for **2-OH** was applied using 4-fluoro-2-hydroxybenzaldehyde. Yield 67%. ^1H NMR (500.1 MHz, $CDCl_3$): 8.54 (d, 2H), 7.60 (t, 2H), 7.30 (d, 2H), 7.14 (t, 2H), 6.97 (t, 1H), 6.61 (dd, 1H), 6.45 (dt, 1H), 3.85 (s, 4H) and 3.73 (s, 2H). ^{13}C NMR (125.8 MHz, $CDCl_3$): 164.54, 162.60, 159.13(d), 158.19, 148.81, 136.91, 130.97, 123.15,

122.29, 118.93, 105.47(d), 104.08 (d), 58.81, 56.21. ^{19}F (470.6 MHz, $CDCl_3$): -114.87. HRMS (ESI) (m/z): $[M+H]^+$calcd. For $C_{19}H_{19}N_3OF$ 324.1505, found 324.1512.

Compound 2-OAc: Acetic anhydride (1.17mL, 12.4 mmols) was added to a solution of compound **2-OH** (0.4g, 1.23mmols) dissolved in pyridine (0.99ml, 12.4mmols). The mixture was left stirred overnight. The crude mixture was diluted with EtOAc and three times washed with 1M HCl and aqueous $NaHCO_3$ solution (3x). The combined organic layer was dried over Na_2SO_4, filtered, and concentrated under reduced pressure. The crude residue was washed three times with cold hexane (5ml) and decanted the hexane to obtain the **2-OAc** as an orange oil (110mg, 24%). ^{1}H NMR (400.35 MHz, $CDCl_3$): 8.54 (d, 2H), 7.69 (m, 2H), 7.66 (d, 2H), 7.45 (m, 1H), 7.17 (m, 2H), 7.00 (m, 2H), 3.82 (s, 4H), 3.61 (s, 2H), 2.25 (s, 3H). ^{13}C {^{1}H} and DEPTQ {^{1}H} NMR (500 MHz, $CDCl_3$): 169.35 (d) 161.72 (m), 149.07, 145.00, 136.54, 133.45 (d), 123.67, 123.58, 122.84, 122.14, 116.58,116.35, 114.79, 114.55, 60.28, 52.17 (d). ^{19}F (376.66 MHz, $CDCl_3$): -117.39. HRMS (ESI) (m/z): $[M+H]^+$ calcd. For $C_{21}H_{21}N_3O_2F$ 336.1614, found 336.1615.

Compound 1-OAc: A similar method described for **2-OAc** was used to synthesize and purify using compound **1-OH**. ^{1}H NMR (500.1 MHz, $CDCl_3$): 8.86 (d, 2H), 8.21 (t, 2H), 7.93 (d, 2H), 7.69 (t, 2H), 7.54 (t, 1H), 6.82 (t, 1H), 6.67 (d, 1H), 4.26 (s, 4H), 3.69 (s, 2H), 2.32 (s, 3H). ^{13}C NMR (125.8 MHz, $CDCl_3$): 168.93, 162.67, 161.70, 159.316, 149.83(d), 148.89, 136.62, 131.55, 131.47, 126.60, 123.17, 122.15, 113.23, 110.27, 60.01, 52.12, 20.79. ^{19}F (470.6 MHz, $CDCl_3$): -114.16. HRMS (ESI) (m/z): $[M+H]^+$ calcd. For $C_{21}H_{21}N_3O_2F$ 336.1618, found 366.1608.

C. NMR and MRI Experiments

^{19}F-NMR studies:

All ^{19}F- NMR experiments were performed on a 9.4 T MHz AVANCEIII NMR spectrometer (Bruker, Germany). Samples were prepped in a 5 mm NMR tube before the ^{19}F-NMR experiment to a final volume of 0.5 mL containing 10% D_2O used for lock. First, the studied fluorinated probe, **1-OAc** or **2-OAc**, was dissolved at a final concentration of 1 M at DMSO, and this solution was used as a stock solution for further dilutions to 3 mM of the probe (**1-OAc** or **2-OAc**) in the studied aqueous solutions. All studied ions were of chloride salt source ($ZnCl_2$, $CaCl_2$, $MgCl_2$, $MnCl_2$, $NiCl_2$ $CoCl_2$, $FeCl_3$, NaCl, and KCl) except for Fe^{2+}, where $FeSO_4$ salt was used. In the studied buffered (10 mM Hepes or 10 mM PBS solutions, as noted), the pH was adjusted by titration with 1M HCl or 1M NaOH solutions. As noted in each experiment, the temperature was controlled to 298 K (25°C) or 310 K (37°C). The ^{19}F-NMR spectra were acquired with a repetition time of 6 sec and 100 scans for 10 min.

For the $t_{1/2}$ determination experiments, a PBS solution of 3 mM **2-OAc** was freshly prepared before the ^{19}F-NMR experiments with an equimolar concentration (3 mM) of the cation of interest and transferred to a 5 mm NMR tube after fast mixing. Following a brief automatic shimming procedure, consecutive ^{19}F-NMR spectra were recorded with a repetition time of 6 sec and 64 scans/ spectrum for 1 hour. The ratio between the integrals of the ^{19}F-NMR peaks of **2-OAc** (-117

ppm) and **2-OH-Zn^{2+}** (-129 ppm) was calculated and plotted over time to determine the $t_{1/2}$, the time at which half of 2-**OAc** was hydrolyzed.

<u>^{19}F-MRI studies</u>:
A phantom composed of six tubes was set where each sample tube contained **2-OAc** (3 mM) in PBS (pH = 7.2), and one of the examined cations at equimolar concentration (Ca^{2+}, Mg^{2+}, Na^{+}, K^{+}, Zn^{2+} or no cation). The phantom was placed in a 9.4 T MRI scanner (Bruker AVANCE III system), and both ^{1}H-MRI and ^{19}F-MRI data were obtained after the temperature was stabilized at 301K. For ^{1}H-MRI a Rapid Acquisition Relaxation Enhanced (RARE) sequence was used with the following parameters: 15 slices of 0.8 mm thickness, FOV: 48×48, Matrix: 256×256, TR/TE: 1000/31 ms, RARE Factor: 8, and a single average to result in a 32 sec time of acquisition. For ^{19}F-MRI, two ^{19}F-MRI data sets were acquired with a RARE sequence where the center frequency O_1 set at -117 ppm (without Zn^{2+} activity) or -129 ppm (following Zn^{2+} activity) with the following parameters: A single slice of 10 mm thickness, FOV: 48×48, Matrix: 32×32, TR/TE: 3000/9 ms, RARE Factor: 8, and 600 averages to result in a 1 hour time of acquisition.

D. X-ray crystallography.
Compound 2-OAc + Zn^{2+} Complex: Solutions of compound **2-OH** and $Zn(ClO_4)_2.6H_2O$ dissolved in methanol were combined, sonicated and left at room temperature for slow evaporation. A single-crystal suitable for X-ray crystallography was obtained.

Compound 1-OAc + Zn^{2+} Complex: Solutions of compound **1-OH** and $Zn(ClO_4)_2.6H_2O$ dissolved in methanol were combined, sonicated and left at room temperature for slow evaporation. A single-crystal suitable for X-ray crystallography was obtained.

Suitable crystals of both compounds immersed in paratone oil were mounted onto the diffractometer under liquid nitrogen stream. Diffraction data were collected on a sealed tube Rigaku Synergy-S dual source diffractometer equipped with Dectris Pilatus3R CdTe 300K detector and microfocus, using Mo-Kα radiation (0.71073 A) at 100(2) K using a nitrogen stream. Data were processed with CrysAlis^{PRO} and the structures were solved by direct methods using the SHELXT[1]. Refinement was performed based on F2 with SHELXL[2] and OLEX2[3]. Hydrogen atoms were assigned isotropic in riding mode. Crystallographic data and refinement parameters are summarized in Supplementary Table S1 and shown in Supporting Figures S1 and S2.

E. Supporting Tables

Table S1. Crystallographic data collection and refinement statistics of compounds **1-OAc + Zn^{2+}** and **2-OAc + Zn^{2+}**.

**Species**	**1-OAc + Zn^{2+}**	**2-OAc + Zn^{2+}**
**CCDC No.**	2366645	2366644
**Formula***	$C_{38}H_{34}F_2N_6O_2Zn_2$+2$ClO_4$	$C_{38}H_{34}F_2N_6O_2Zn_2$+2$ClO_4$+solvent
**Molecular weight**	974.35	974.39
**Crystal system**	Triclinic	Tetragonal
**Space group**	*P-1*	*$I4_1/a$*
**Crystal size (mm)**	0.370×0.328×0.087	0.157×0.038×0.033
**Crystal color and shape**	Colorless Plate	Colorless needle
**Temperature (K)**	100	100
**Wavelength (Å)**	0.71073	0.71073
**a (Å)**	10.3739(2)	26.4186(6)
**b (Å)**	19.7493(4)	26.4186(6)
**c (Å)**	20.0456(4)	11.4693(4)
**α (º)**	70.874(2)	90
**β (º)**	80.423(2)	90
**γ (º)**	78.725(2)	90
**Volume ($Å^3$)**	3782.0(2)	8004.9(5)
**Z**	4	8
**r_{calcd} ($g \cdot cm^{-1}$)**	1.711	1.407
**μ (mm^{-1})**	1.489	1.617
**No. of reflections (unique)**	18721(18721)	21304 (4395)
**R_{int}**	0.0493	0.0449
**Completeness to θ (%)**	99.8	99.8
**θ max**	28.282	27.101
**Data / restraints / parameters**	18721 / 0 / 1082	4395 / 0 / 271
**Goodness-of-fit on F^2**	1.028	1.033
**Final R_1 and wR_2 indices [I>2s(I)]**	0.0527, 0.1342	0.0372, 0.0904
**R_1 and wR_2 indices (all data)**	0.0728, 0.1434	0.0481, 0.0948
Largest electron density peak and largest hole	2.123 and -0.808	0.629 and -0.300

Table S2. Cell culture medium DMEM content.

Components in DMEM used	gr/ liter
Calcium Chloride	0.2
Ferric Nitrate • $9H_2O$	0.0001
Magnesium Sulfate (anhydrous)	0.09767
Potassium Chloride	0.4
Sodium Bicarbonate	3.7
Sodium Chloride	6.4
Sodium Phosphate Monobasic (anhydrous)	0.109
L-Arginine • HCl	0.084
L-Cystine • 2HCl	0.0626
L-Glutamine	0.584
Glycine	0.03
L-Histidine • HCl • H_2O	0.042
L-Isoleucine	0.105
L-Leucine	0.105
L-Lysine • HCl	0.146
L-Methionine	0.03
L-Phenylalanine	0.066
L-Serine	0.042
L-Threonine	0.095
L-Tryptophan	0.016
L-Tyrosine • 2Na • $2H_2O$	0.10379
L-Valine	0.094
Choline Chloride	0.004
Folic Acid	0.004
myo-Inositol	0.0072
Niacinamide	0.004
D-Pantothenic Acid (hemicalcium)	0.004
Pyridoxine • HCl	0.004
Riboflavin	0.0004
Thiamine • HCl	0.004
D-Glucose	1
Phenol Red • Na	0.0159
Pyruvic Acid • Na	0.11

F. Supporting Figures (S1-S32).

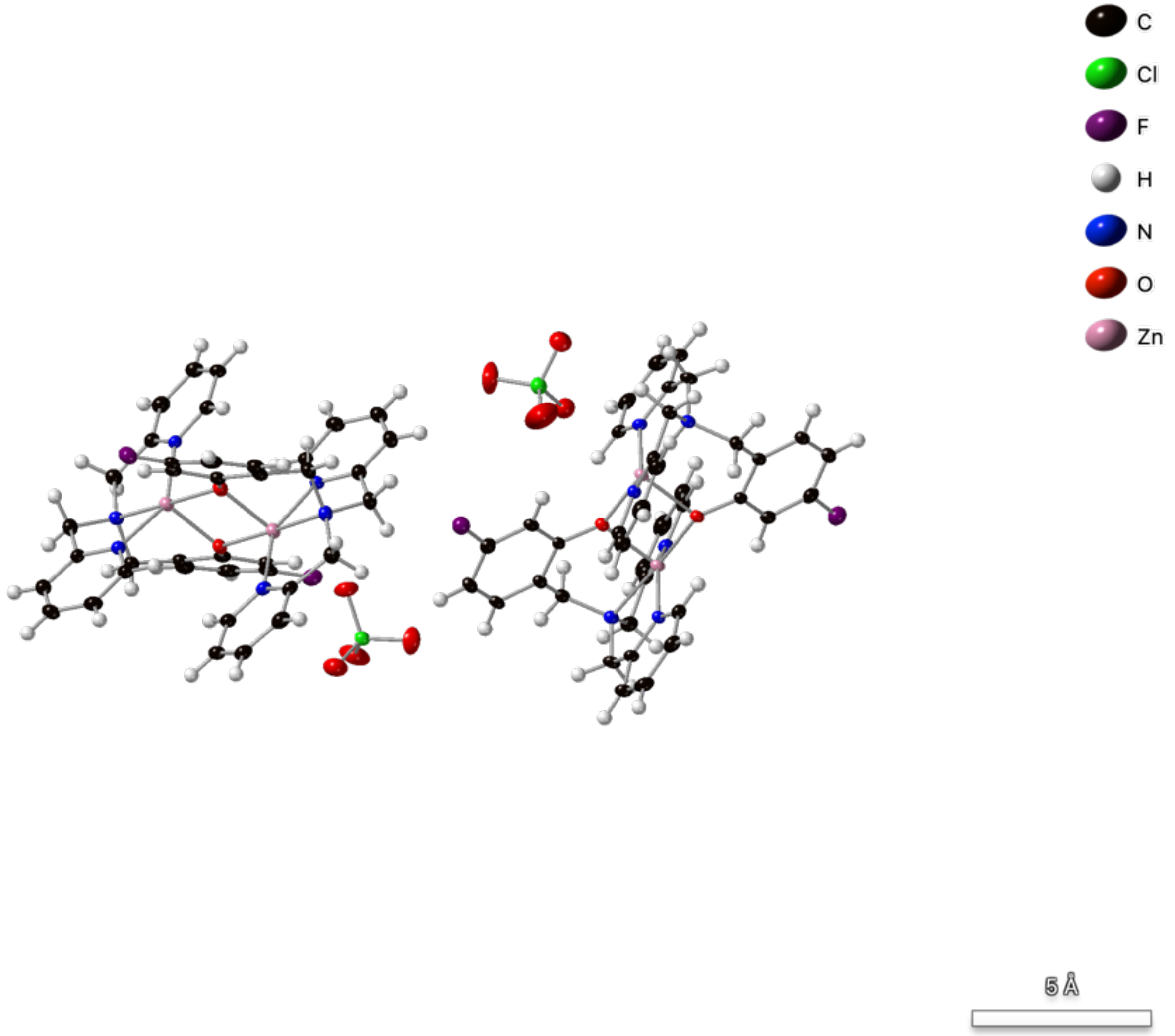

Figure S1. ORTEP representation of 1-OAc+ Zn^{2+}. The thermal ellipsoids are presented in a probability level of 50 %. Hydrogens are presented as spheres. (C: black, Cl: green, F: purple, H: white, N: blue, O: red and Zn: pink)

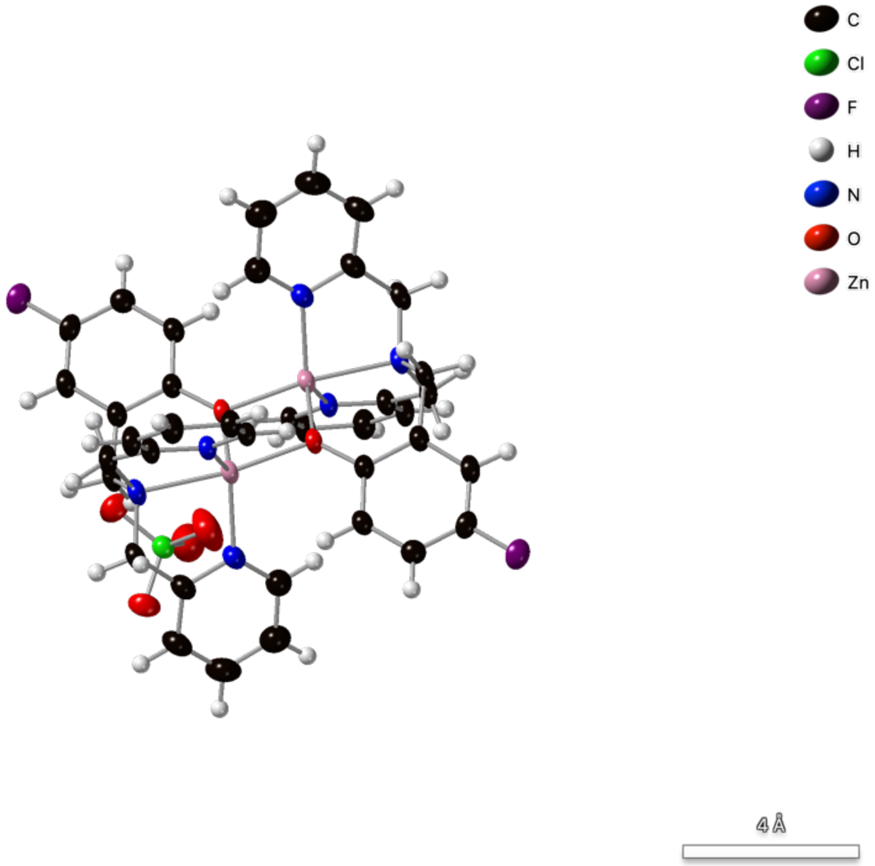

Figure S2. ORTEP representation of **2-OAc+ Zn^{2+}**. The thermal ellipsoids are presented in a probability level of 50 %. Hydrogens are presented as spheres. (C: black, Cl: green, F: purple, H: white, N: blue, O: red and Zn: pink)

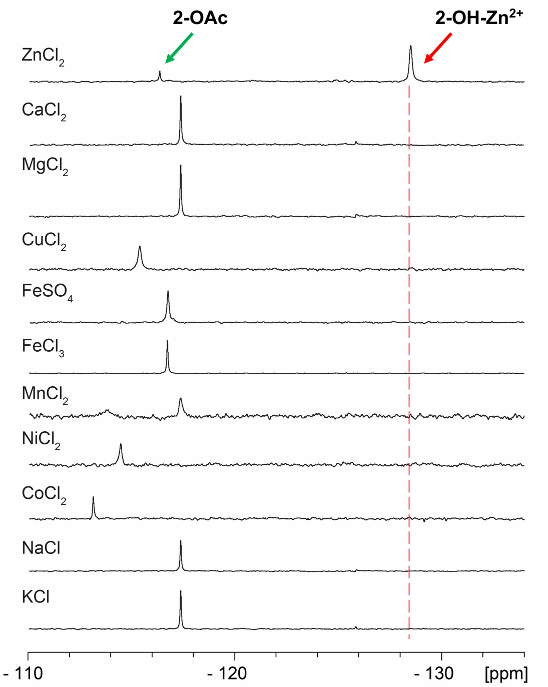

Figure S3. ^{19}F NMR spectra of 2-OAc in the presence of different ions in HEPES buffered solution (pH=7.2). The chemical shift at -129 ppm represents the **2-OH-Zn^{2+}** complex. The ^{19}F-NMR peak at -117 ppm represents the **2-OAc** in the solution. In the presence of some ions, the chemical shift of **2-OAc** was found to be broader or shifted most probably as a result of the paramagnetic properties of the ions (i.e., Cu^{2+}, Fe^{2+}, Fe^{3+}, Mn^{2+}, Ni^{2+}, or Co^{2+}). In none of the studied solutions, except the one that contained Zn^{2+}, no peak at -129 ppm could be detected, emphasizing the specificity of the method to detect Zn^{2+}. An 11.7 T NMR spectrometer was used to acquire the data.

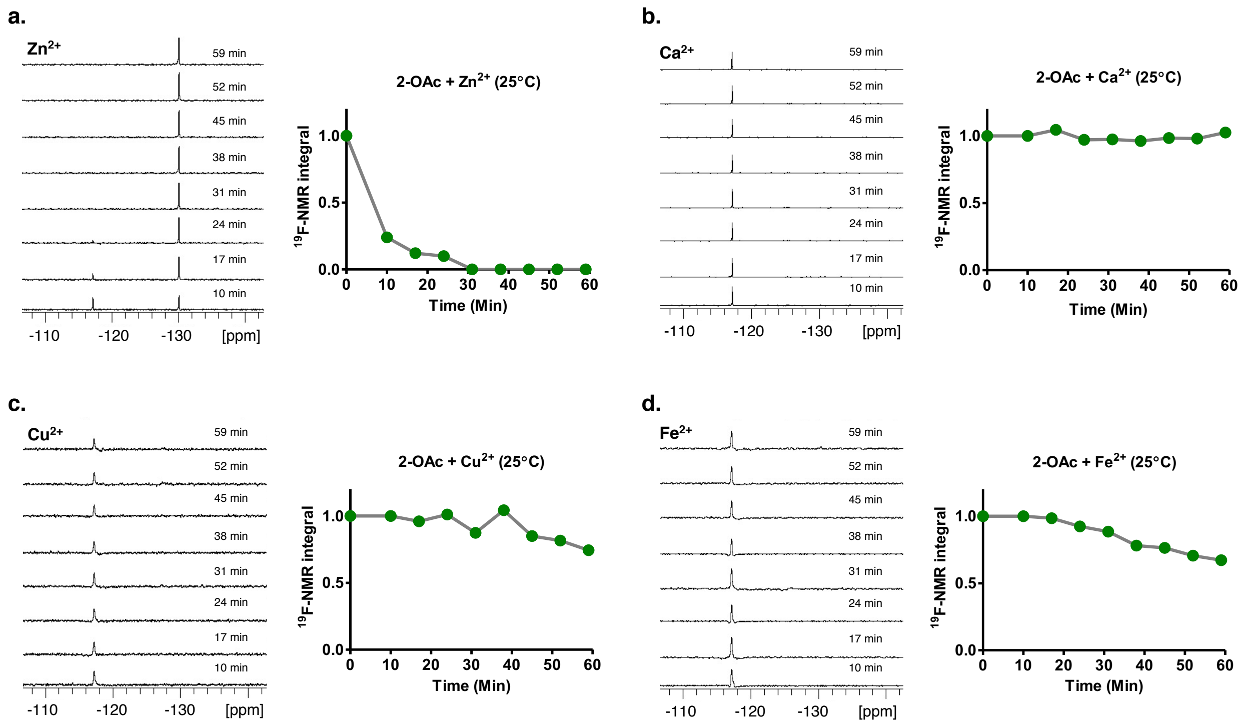

Figure S4. The full experimental data set used for Figure 3. Real-time ^{19}F NMR spectra of **2-OAc** in the presence of Zn^{2+} (a), Ca^{2+} (b), Cu^{2+} (c), and Fe^{2+} (d). The chemical shift of **2-OAc** is -117 ppm and the chemical shift of **2-OH-Zn**$^{2+}$ complex is shown at -129 ppm. Plots with green dots represent the value of the ^{19}F-NMR peak integral at -117 ppm (non-hydrolyzed **2-OAc**). The pH of the studied solution was 7.3 (HEPES Buffered solution), and the temperature was set to 25 °C. An 11.7 T NMR spectrometer was used to acquire the data.

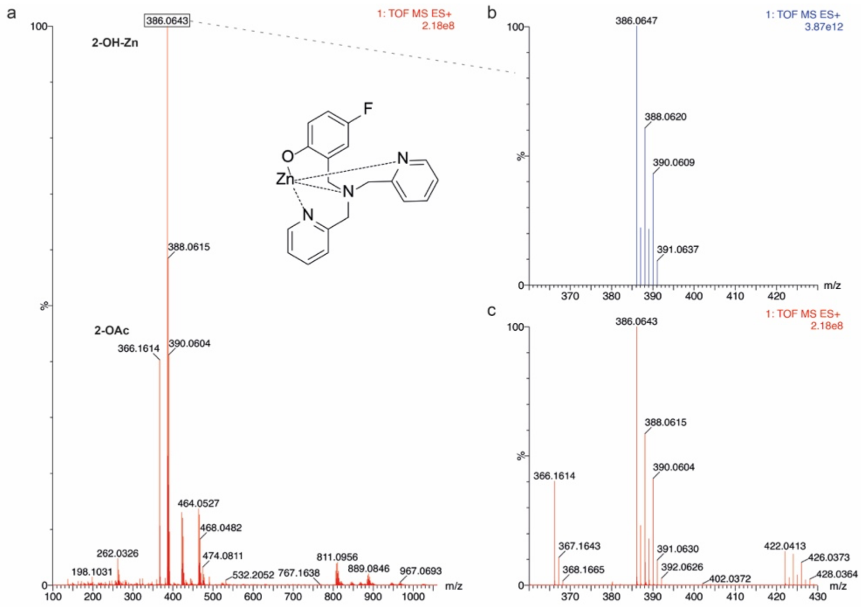

Figure S5. Mass spectroscopy analysis for aqueous solution of 2-OAc in the presence of $ZnCl_2$. (a) Full range spectrum showing the mass of the **2-OH-Zn^{2+}** complex at 386.0643 m/z. (b) The predicted (simulated) isotopic mass distribution of the **2-OH-Zn^{2+}** complex. (c) The experimental (observed) isotopic mass distribution of the **2-OH-Zn^{2+}** complex.

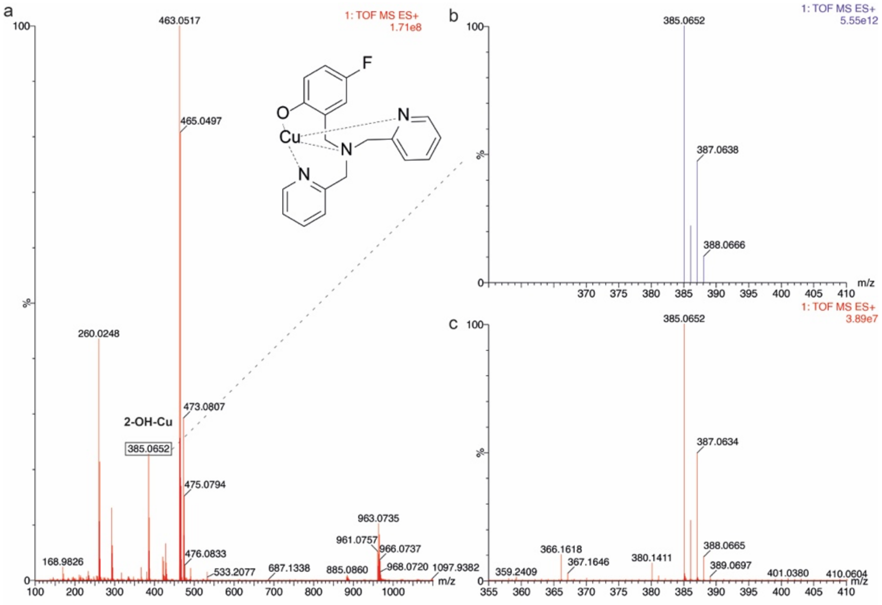

Figure S6. Mass spectroscopy analysis for aqueous solution of 2-OAc in the presence of $CuCl_2$. (a) Full range spectrum showing the mass of the **2-OH-Cu^{2+}** complex at 385.0652 m/z. (b) The predicted (simulated) isotopic mass distribution of the **2-OH-Cu^{2+}** complex. (c) The experimental (observed) isotopic mass distribution of the **2-OH-Cu^{2+}** complex.

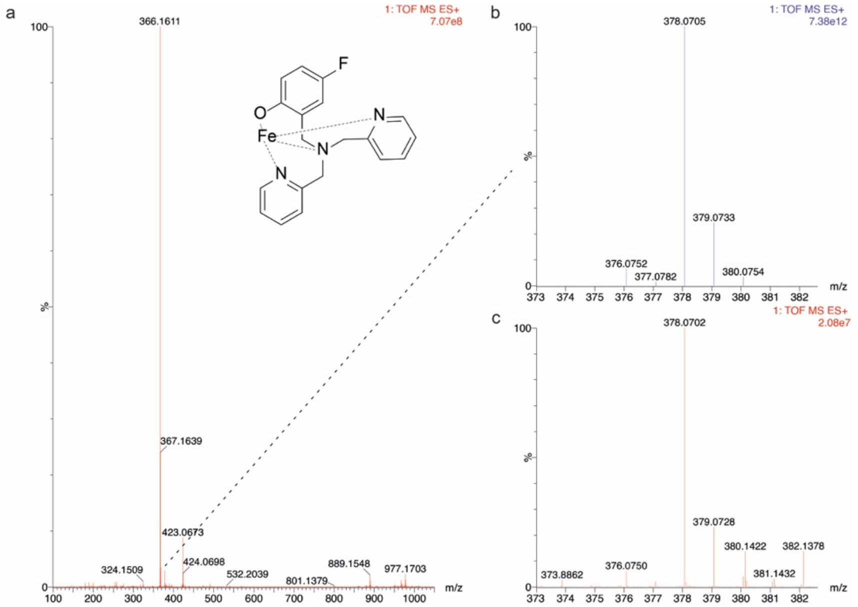

Figure S7. Mass spectroscopy analysis for aqueous solution of 2-OAc in the presence of $FeSO_4$. (a) Full range spectrum showing only traces of the mass of the **2-OH-Fe^{2+}** complex at 378.0702 m/z. (b) The predicted (simulated) isotopic mass distribution of the **2-OH-Fe^{2+}** complex. (c) The experimental (observed) isotopic mass distribution of the **2-OH-Fe^{2+}** complex.

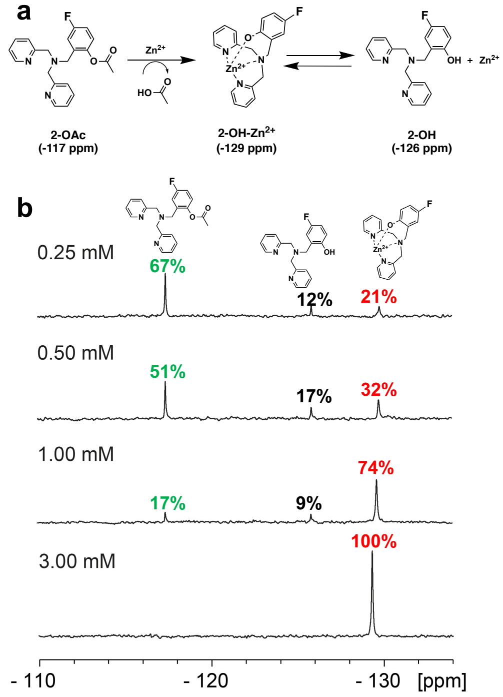

Figure S8. Zn^{2+}-induced hydrolysis of 2-OAc at different Zn^{2+} concentrations. (a) The chemical reaction of the hydrolysis of **2-OAc** (-117 ppm at the ^{19}F-NMR spectrum) as catalyzed by Zn^{2+} to form **2-OH-Zn^{2+}** complex (-129 ppm at the ^{19}F-NMR spectrum), which releases some of the Zn^{2+} (K_d dependence) to obtain free **2-OH** (-126 ppm at the ^{19}F-NMR spectrum). (b) ^{19}F-NMR spectra of aqueous solutions of **2-OAc** (3 mM in HEPES buffered solution, pH=7.2) in the presence of different concentrations of Zn^{2+} as noted (0.25 mM, 0.5 mM, 1.0 mM, and 3.0 mM). NMR measurements were performed on an 11.7 T NMR spectrometer, and the 19F-NMR spectra were acquired 45 min after mixing 3 mM **2-OAc** with $ZnCl_2$ solution to obtain the noted Zn^{2+} concentration.

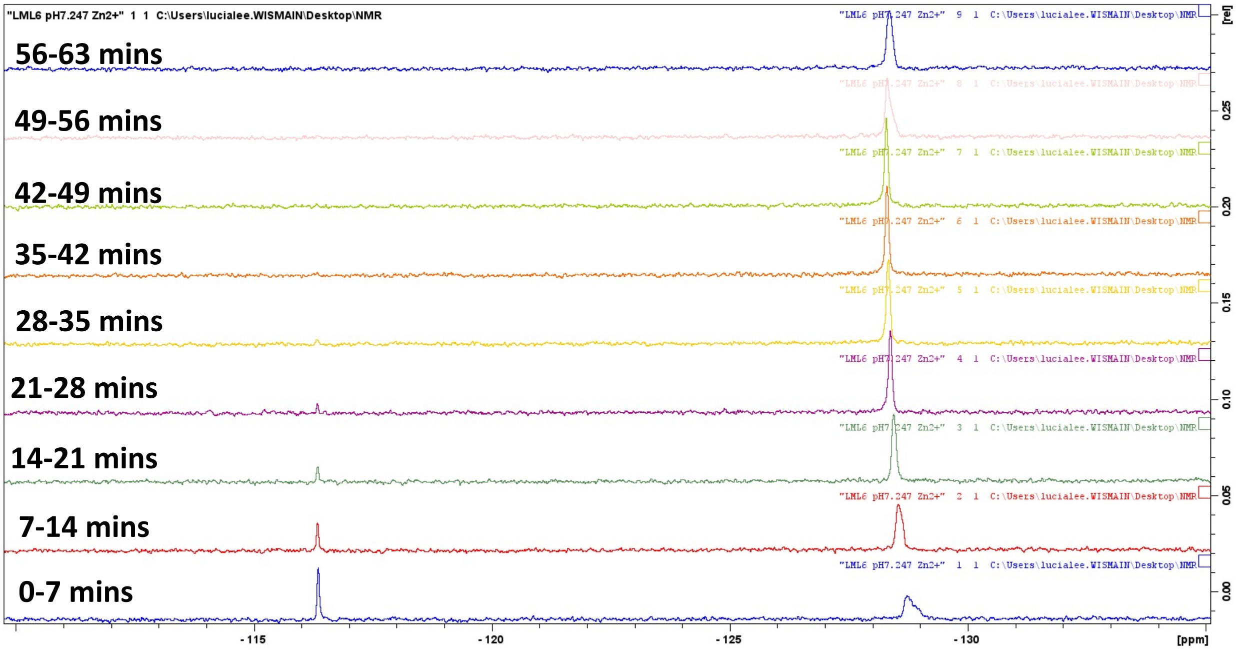

Figure S9. Real-time ^{19}F NMR spectra of 2-OAc in the presence of Zn^{2+} at pH=7.2 and 37 ºC. The chemical shift of **2-OAc** is -117 ppm, and the chemical shift of **2-OH-Zn^{2+}** complex is shown at -129 ppm. An 11.7 T NMR spectrometer was used to acquire the data.

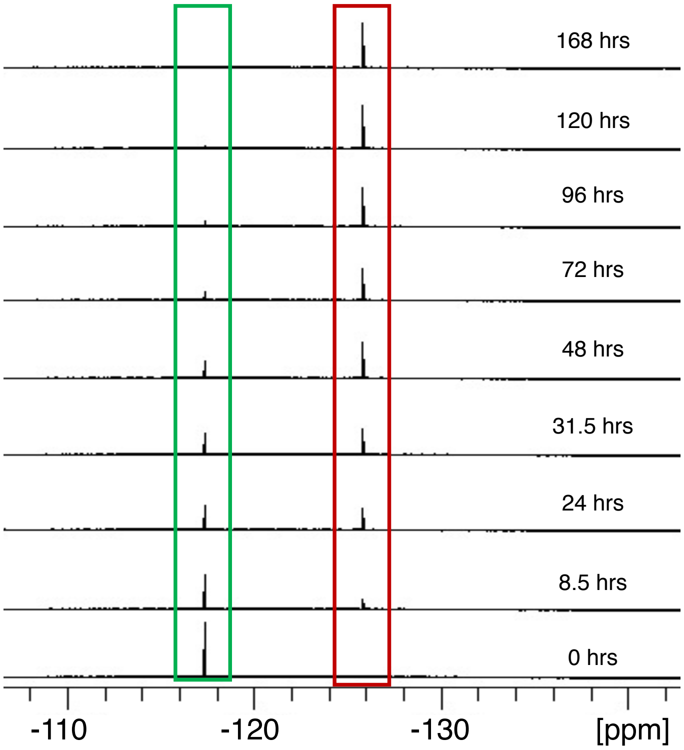

Figure S10. Real-time ^{19}F NMR spectra of 2-OAc in the presence of Ca^{2+} at pH=7.2. The chemical shift of **2-OAc** is -117 ppm, and one of the hydrolyzed compound **2-OH** (without a complex with cation) is shown at -125.7 ppm. An 11.7 T NMR spectrometer was used to acquire the data at 25ºC.

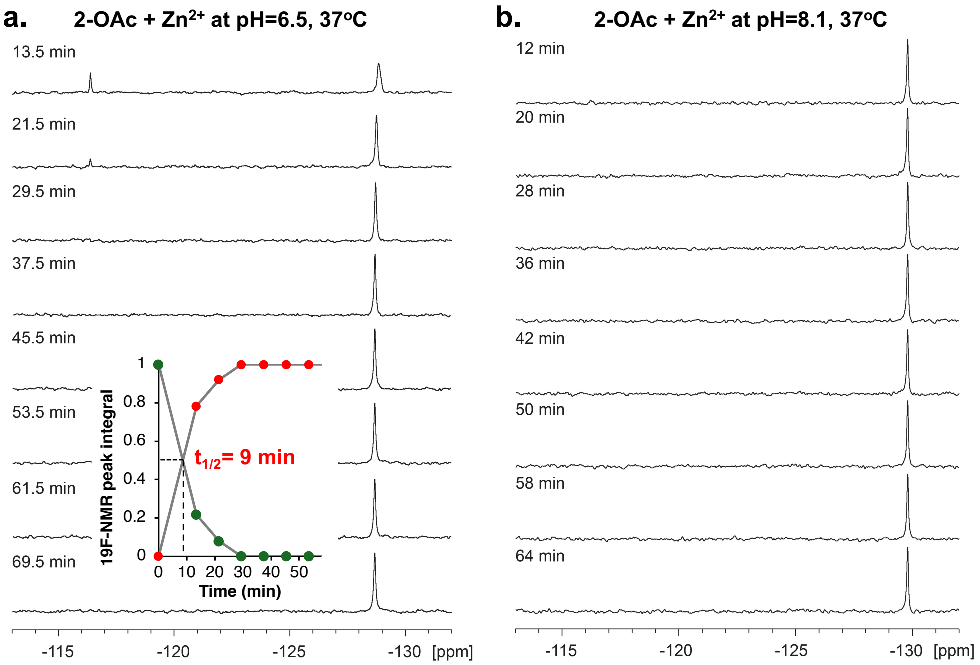

Figure S11. Real-time ^{19}F-NMR spectra of 2-OAc in the presence of Zn^{2+} at acidic and basic pH at physiological temperature (37 ^{o}C). (a) Longitudinal measurements of ^{19}F NMR spectra of **2-OAc** in the presence of Zn^{2+} at pH=6.5 at 37°C. The inset plot at the bottom left shows the ^{19}F-NMR peaks integrals of the two peaks (**2-OAc** at -117 ppm and **2-OH-Zn^{2+}** complex at -129 ppm) with the estimated $t_{1/2}$ of the **2-OAc** hydrolysis ($t_{1/2}$=9 min) An 11.7 T NMR spectrometer was used to acquire the data. (b) Longitudinal measurements of ^{19}F-NMR spectra of **2-OAc** in the presence of Zn^{2+} at pH=8.1 at 37°C. Note that at the first ^{19}F-NMR spectrum acquired (12 min after mixing), there was only one peak of the **2-OH-Zn^{2+}** complex a(t -129 ppm) observed with no evidence of the starting material **2-OAc**. Thus, the $t_{1/2}$ of the **2-OAc** hydrolysis at this pH was too fast to be determined by ^{19}F-NMR.

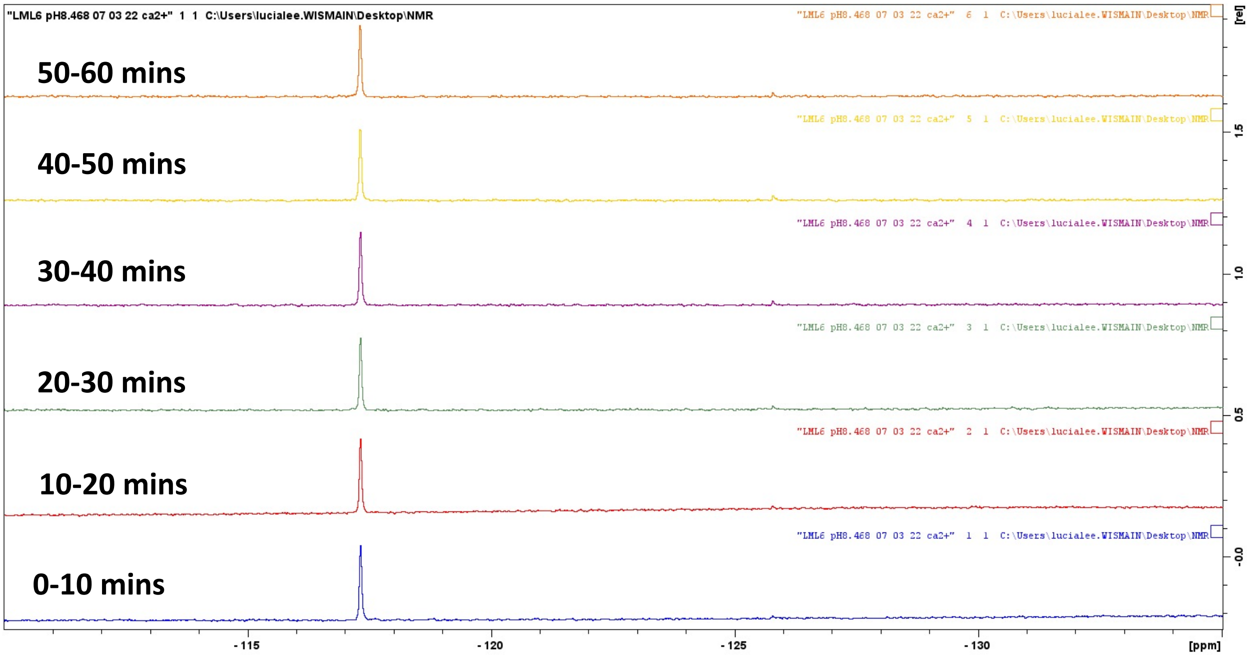

Figure S12. Real-time ^{19}F NMR spectra of 2-OAc in the presence of Ca^{2+} at pH=8.5 (25 ^{o}C). The chemical shift of **2-OAc** is -117 ppm. An 11.7 T NMR spectrometer was used to acquire the data.

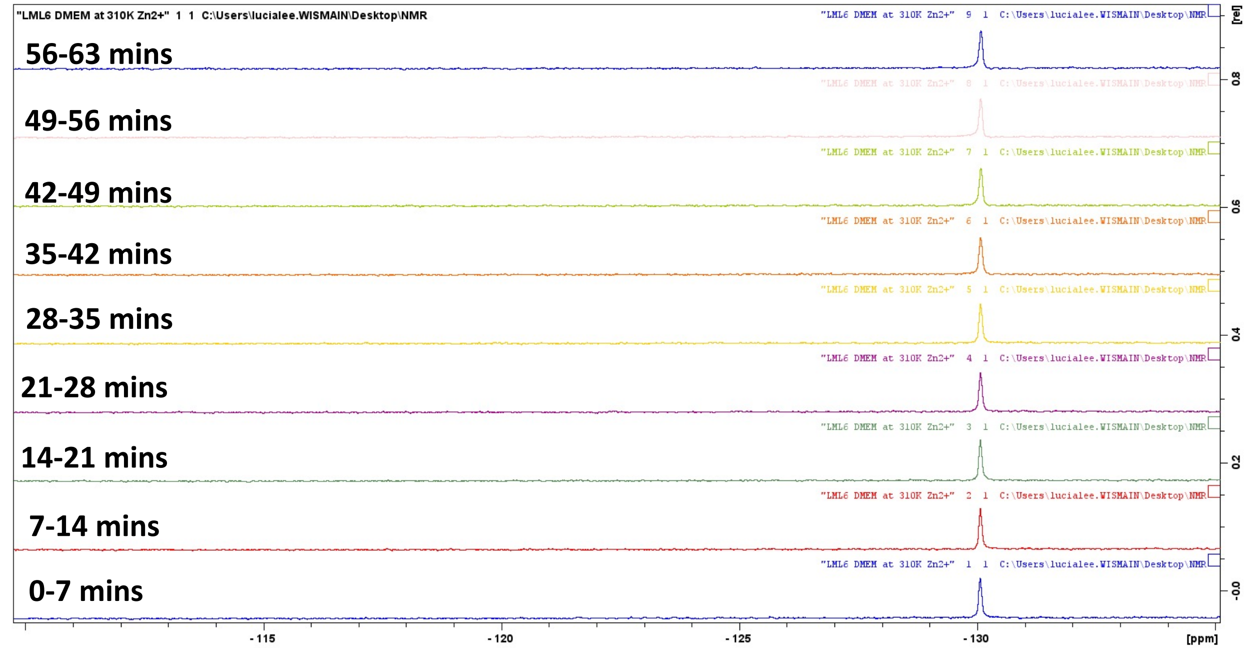

Figure S13. Real-time ^{19}F NMR spectra of 2-OAc in the presence of Zn^{2+} at DMEM+10% FBS solution at 37°C. The chemical shift at -129 ppm represents the **2-OH-Zn^{2+}** complex- no evidence for the existence of **2-OAc** in the solution (peak at -117 ppm). An 11.7 T NMR spectrometer was used to acquire the data.

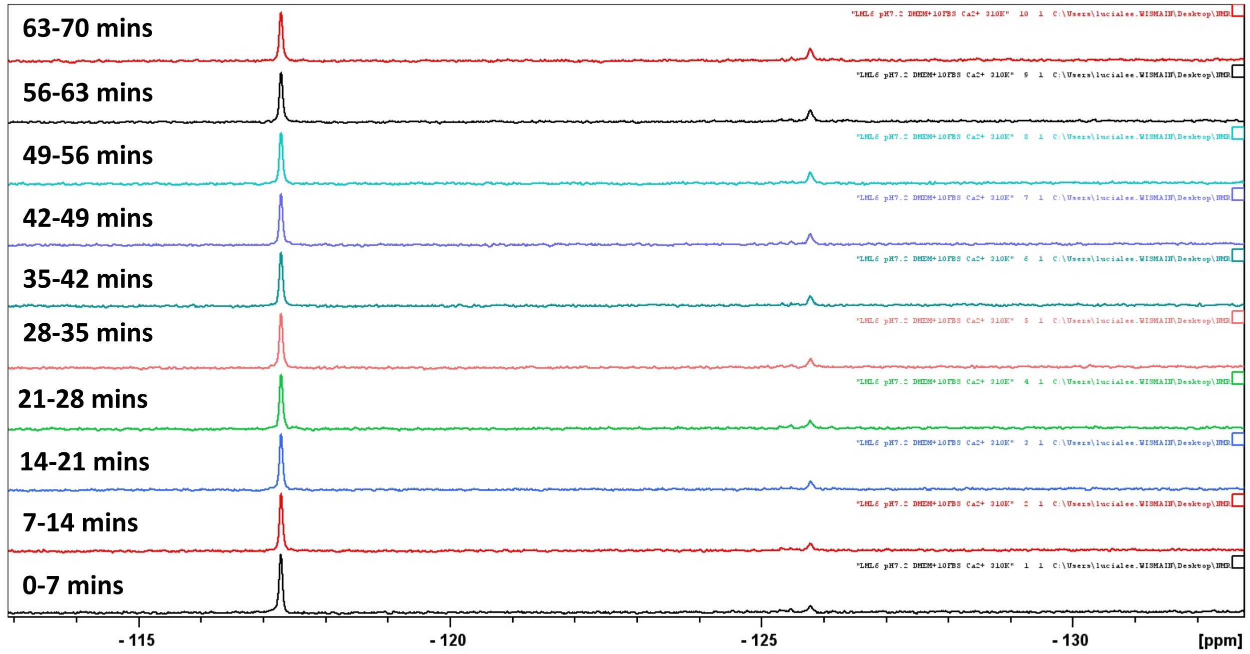

Figure S14. Real-time ^{19}F NMR spectra of 2-OAc in DMEM+10% FBS solution at 37°C. The chemical shift of **2-OAc** is -117 ppm. Traces of naturally hydrolyzed **2-OH** (without a complex with cation) are shown at -125.7 ppm. An 11.7 T NMR spectrometer was used to acquire the data.

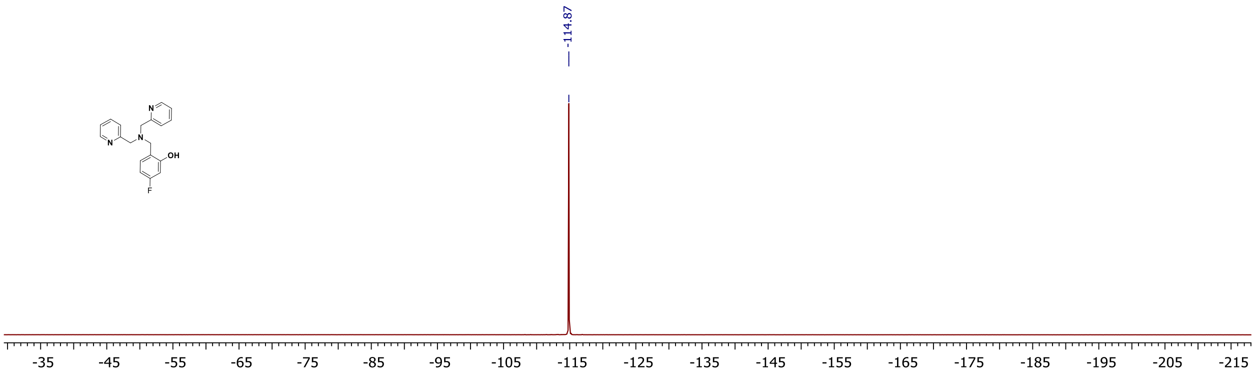

Figure S15. ^{19}F NMR (470.07 MHz, $CDCl_3$, 25 °C) spectrum of compound **1-OH.**

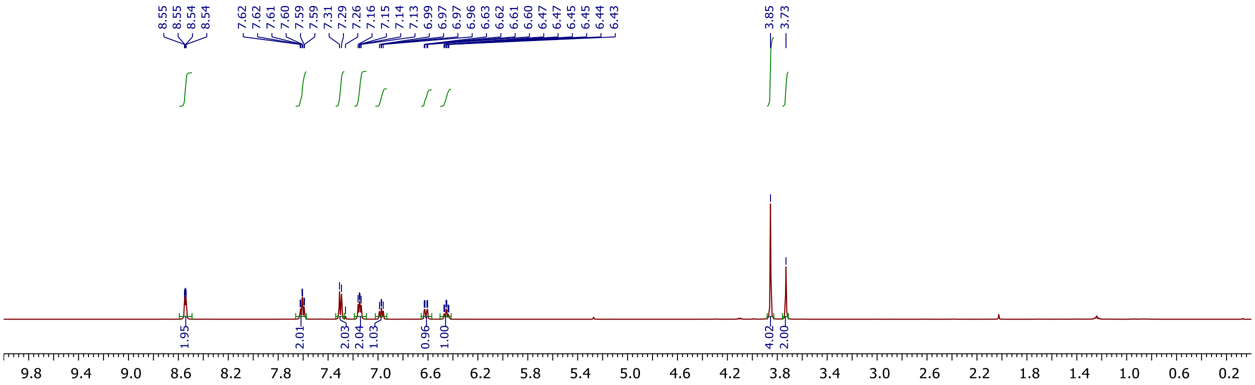

Figure S16. ^{1}H NMR (500.08 MHz, $CDCl_3$, 25 °C) spectrum of compound **1-OH.**

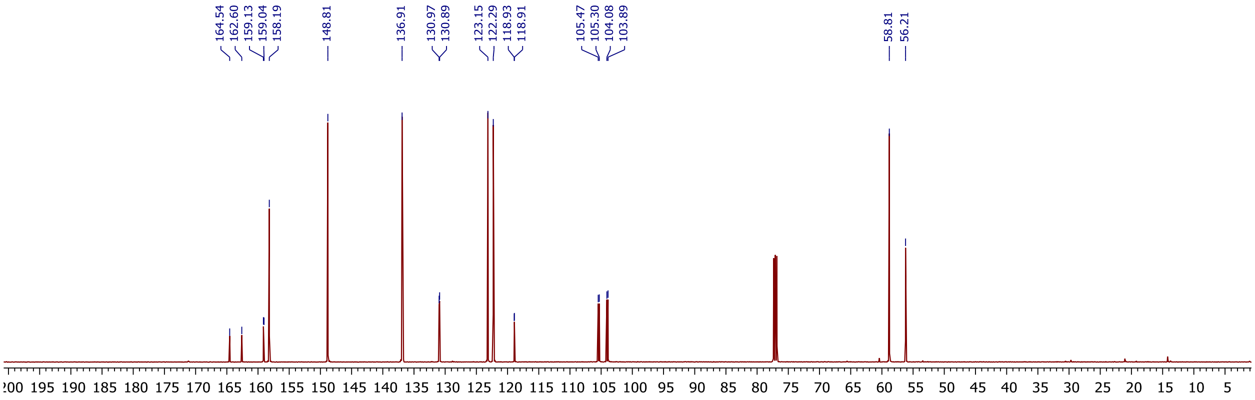

Figure S17. ^{13}C NMR (125.76 MHz, $CDCl_3$, 25 °C) spectrum of compound **1-OH.**

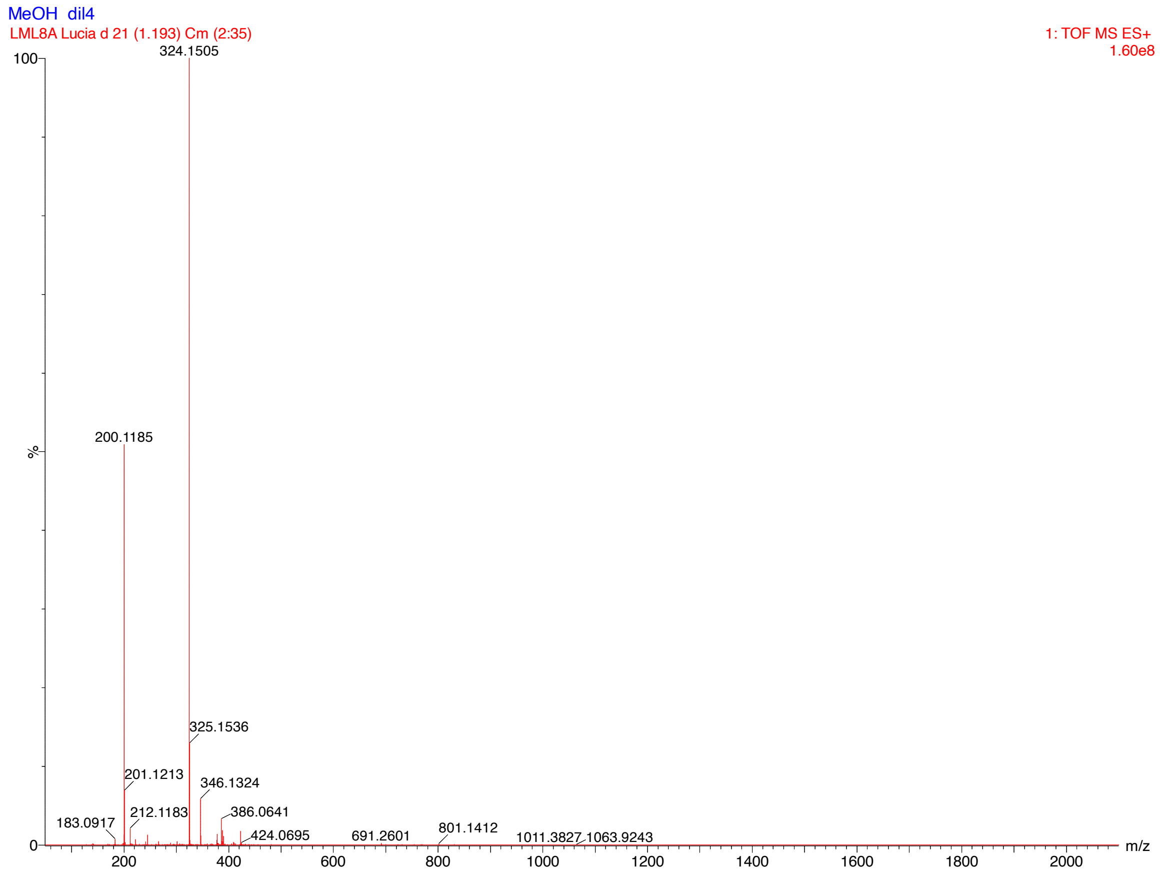

Figure S18. HRMS (ESI) spectrum of compound **1-OH.**

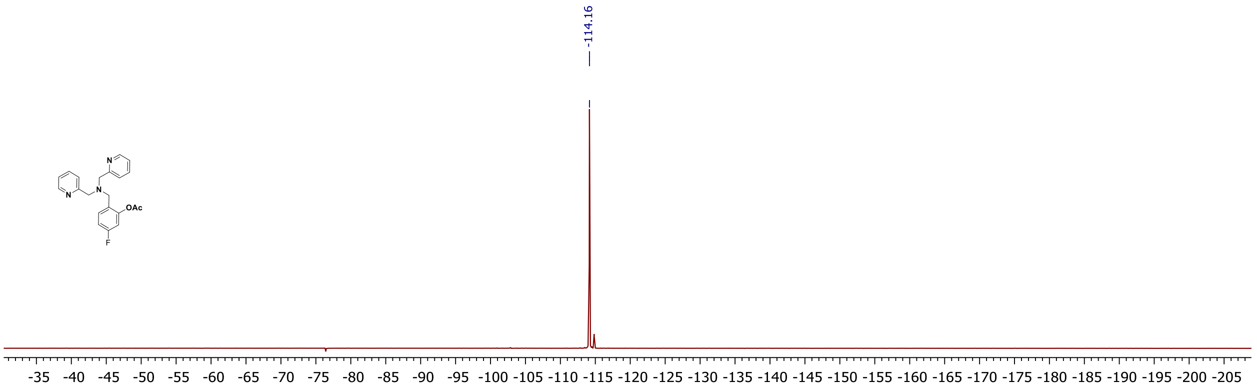

Figure S19. ^{19}F NMR (470.07 MHz, $CDCl_3$, 25 °C) spectrum of compound **1-OAc.**

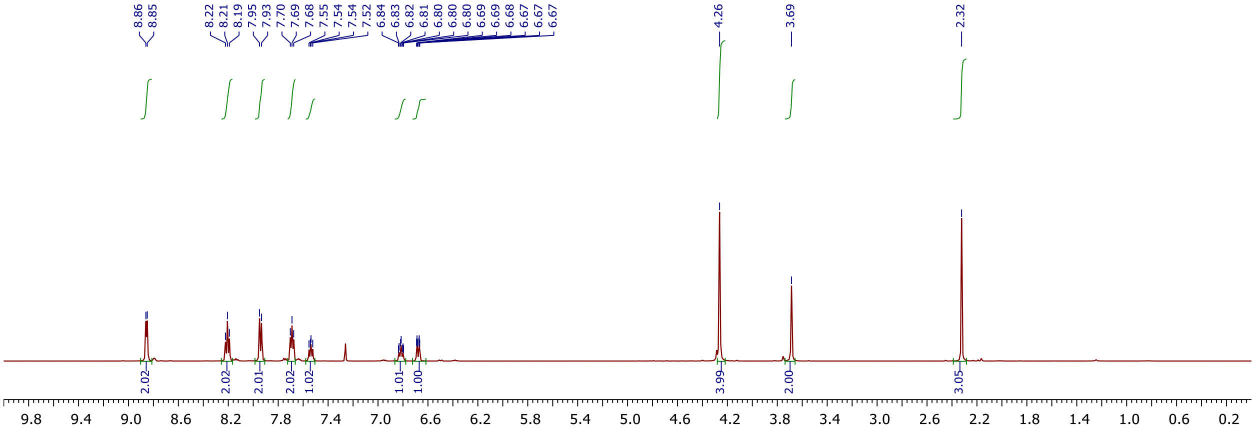

Figure S20. ^{1}H NMR (500.08 MHz, $CDCl_3$, 25 °C) spectrum of compound **1-OAc.**

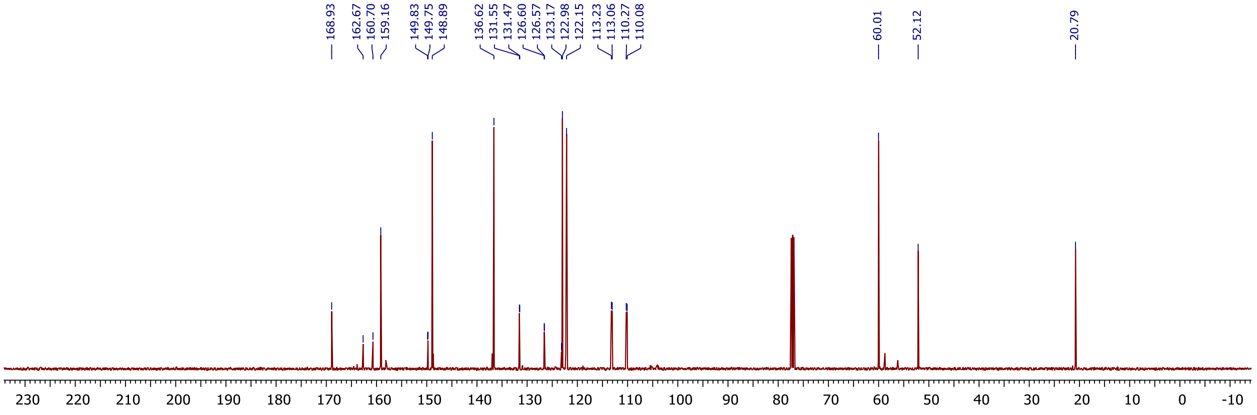

Figure S21. ^{13}C NMR (125.76 MHz, $CDCl_3$, 25 °C) spectrum of compound **1-OAc.**

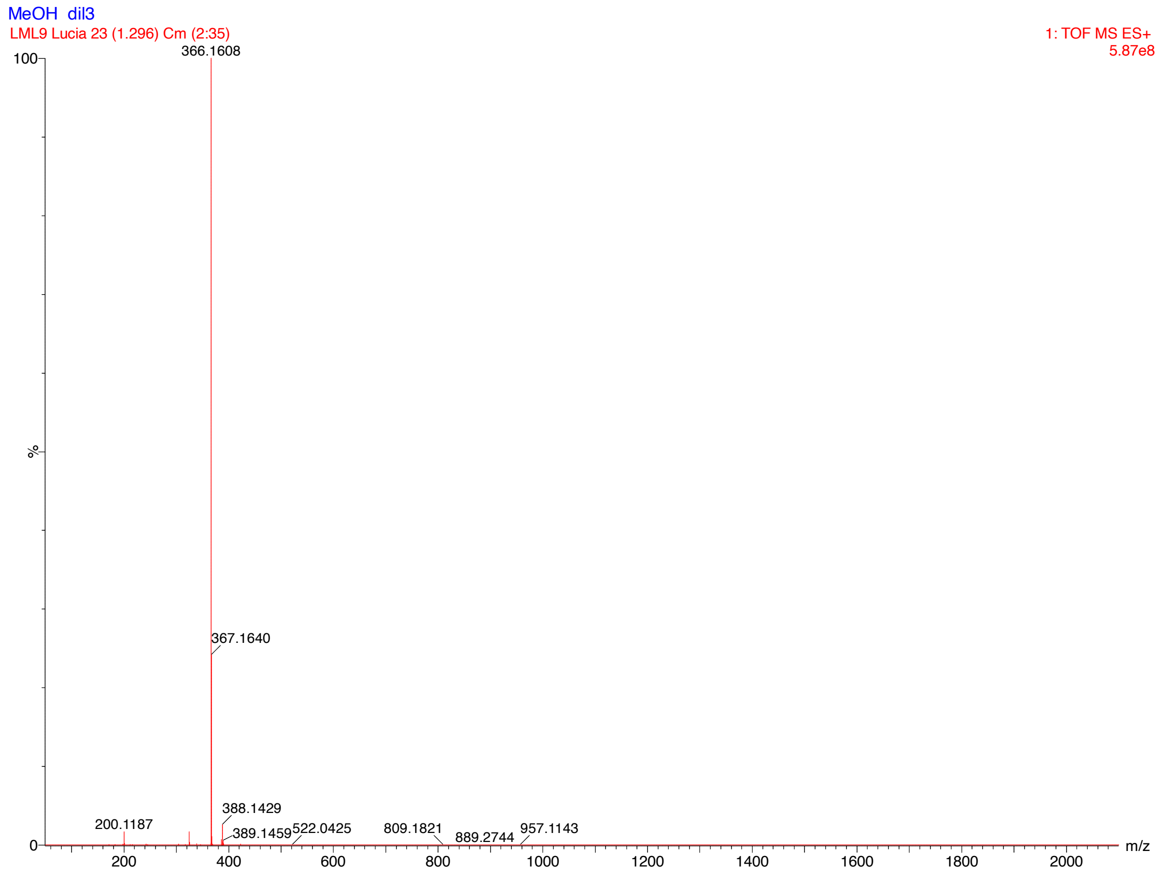

Figure S22. HRMS (ESI) spectrum of compound **1-OAc.**

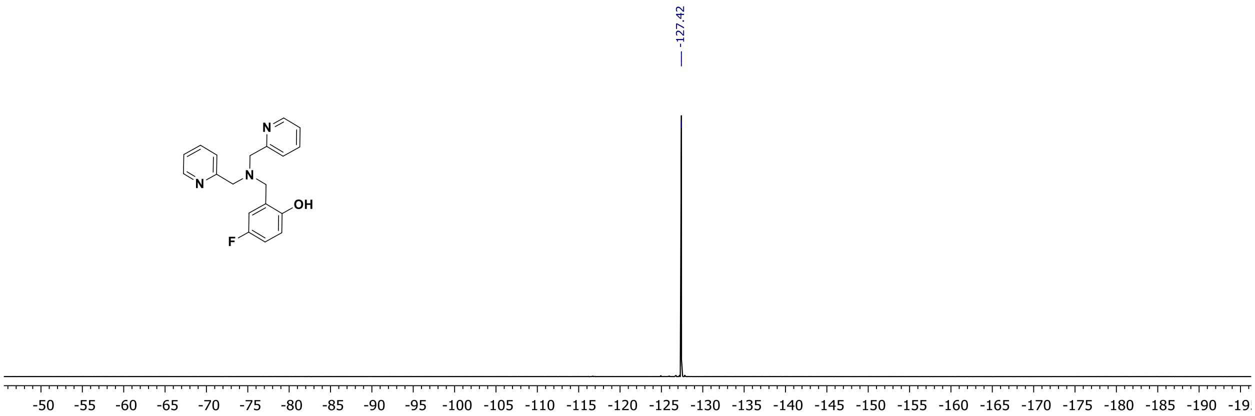

Figure S23. ^{19}F NMR (376.66 MHz, $CDCl_3$, 25 °C) spectrum of compound **2-OH**.

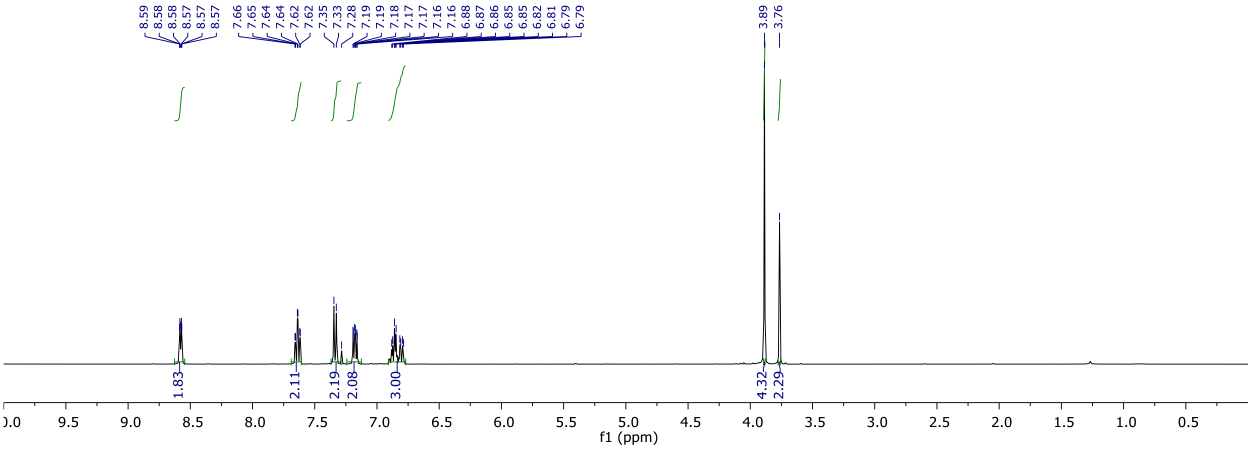

Figure S24. ^{1}H NMR (400.35 MHz, $CDCl_3$, 25 °C) spectrum of compound **2-OH.**

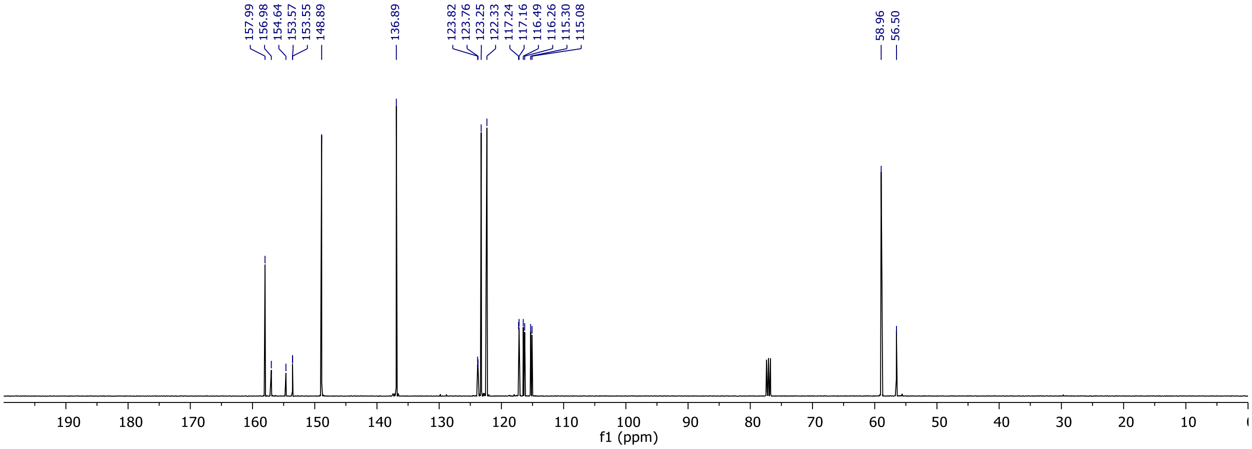

Figure S25. ^{13}C{^{1}H} NMR (100.67 MHz, $CDCl_3$, 25 °C) spectrum of compound **2-OH.**

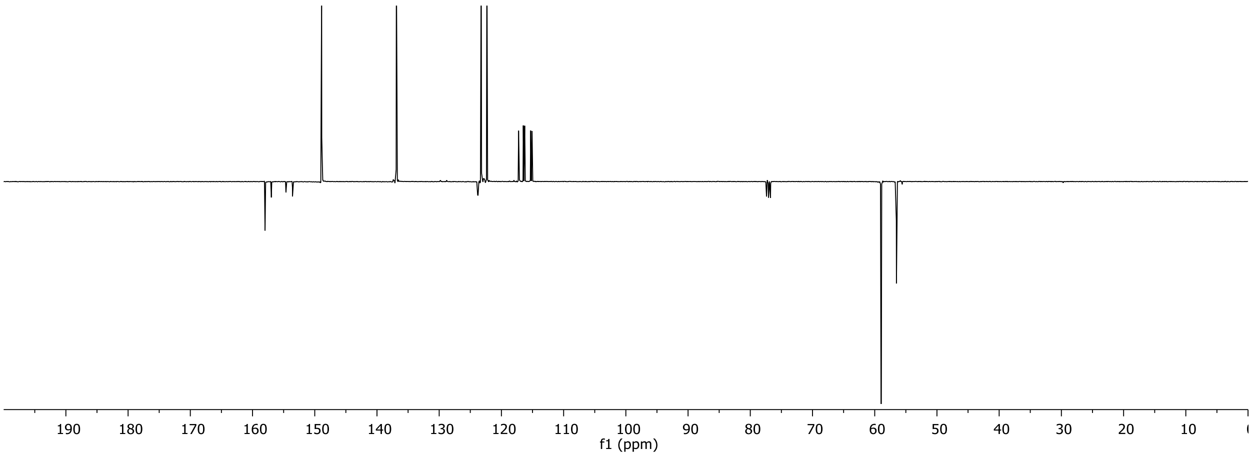

Figure S26: DEPTQ{^{1}H} NMR (100.67 MHz, $CDCl_3$, 25 °C) spectrum of compound **2-OH**.

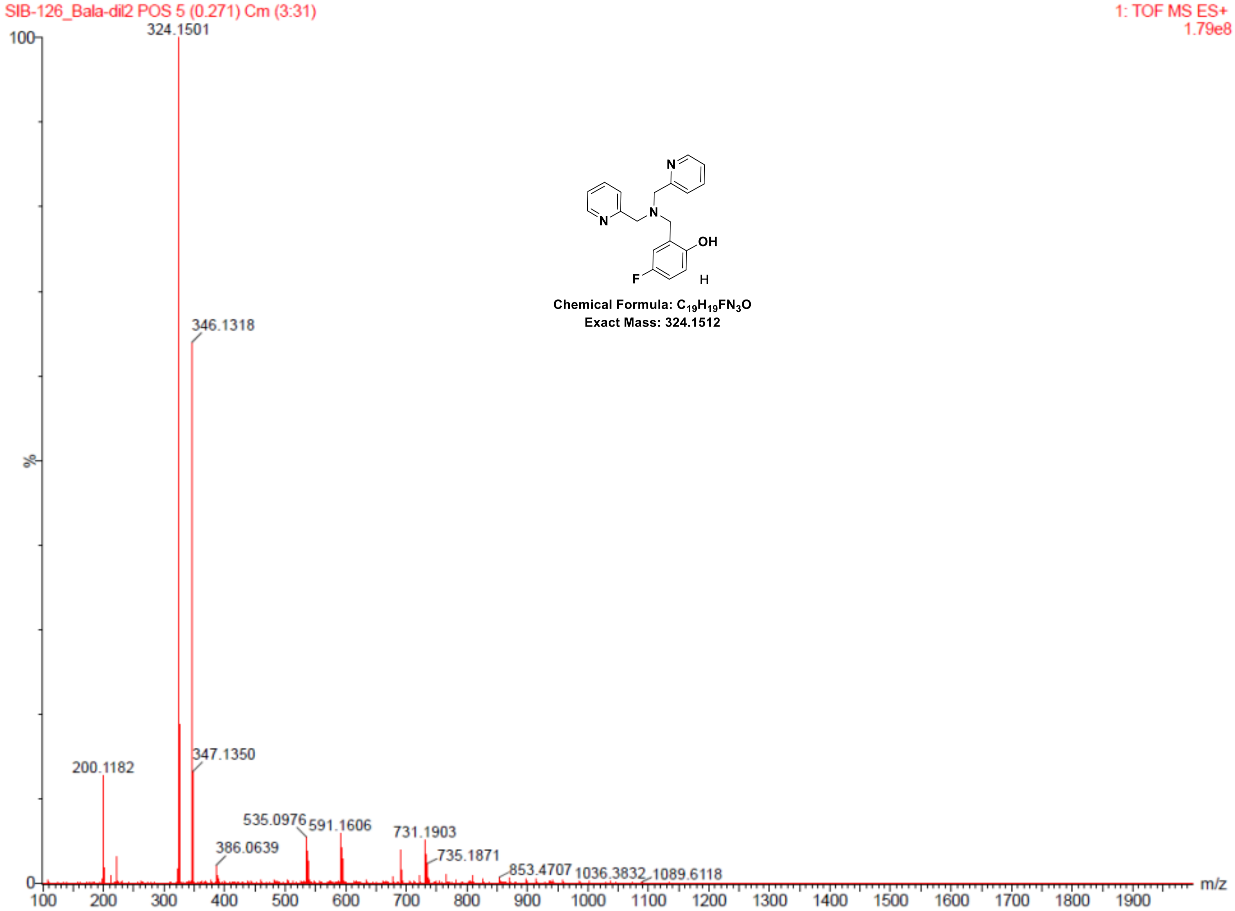

Figure S27. HRMS (ESI) spectrum of compound **2-OH**.

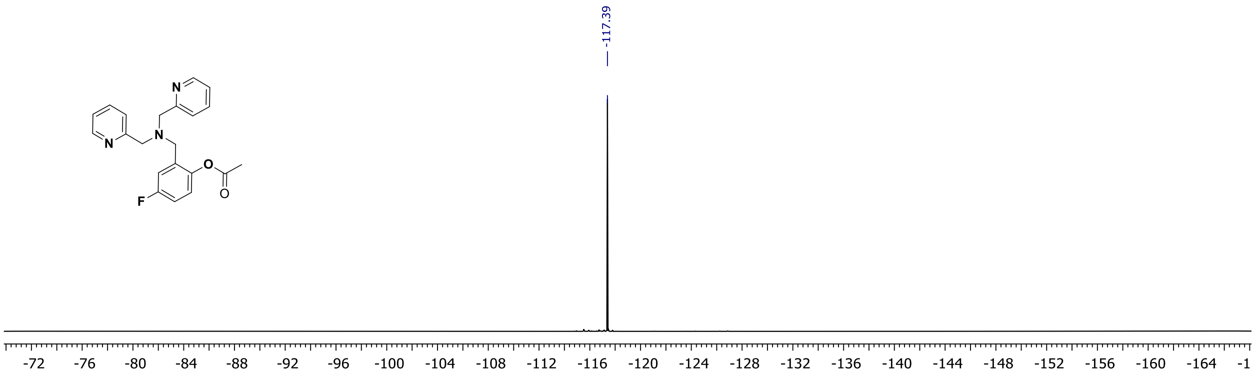

Figure S28. ^{19}F NMR (376.66 MHz, $CDCl_3$, 25 °C) spectrum of compound **2-OAc**.

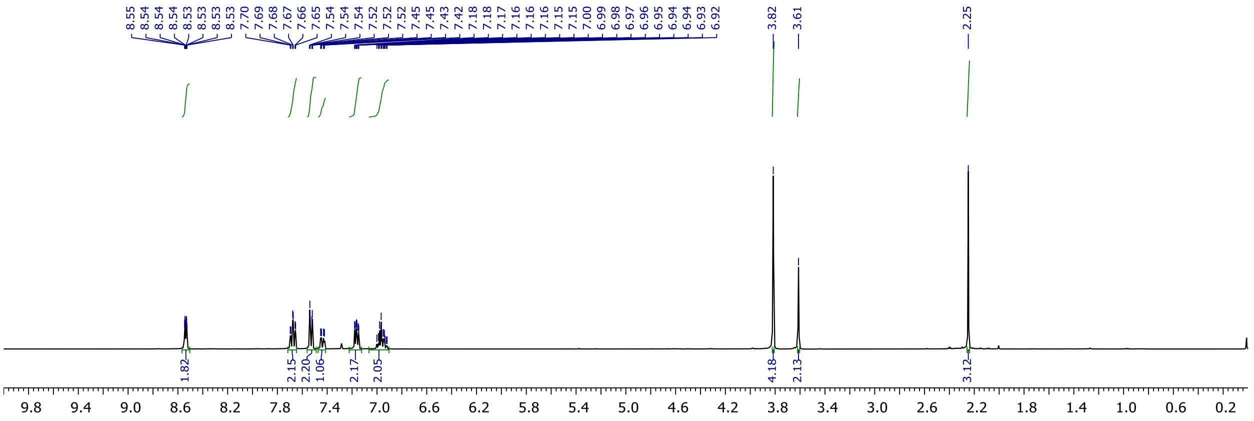

Figure S29.^{1}H NMR (400.35 MHz, $CDCl_3$, 25 °C) spectrum of compound **2-OAc**.

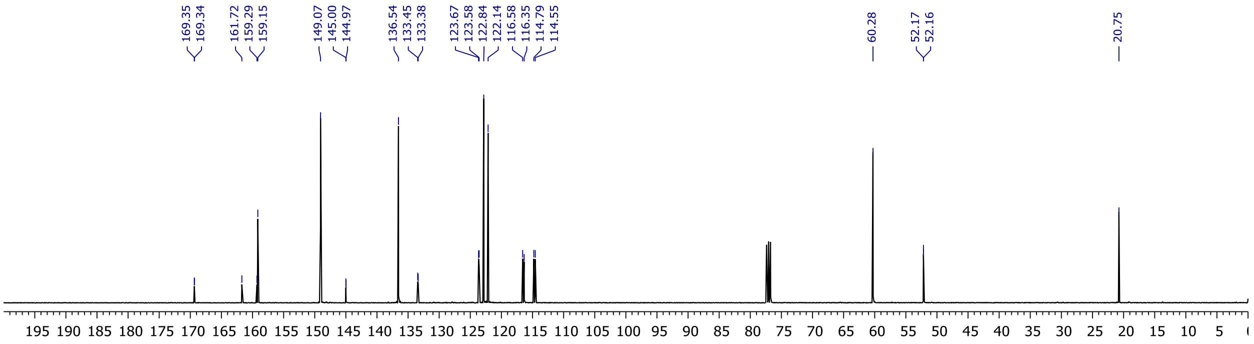

Figure S30. ^{13}C{^{1}H} NMR (100.67 MHz, $CDCl_3$, 25 °C) spectrum of compound **2-OAc**.

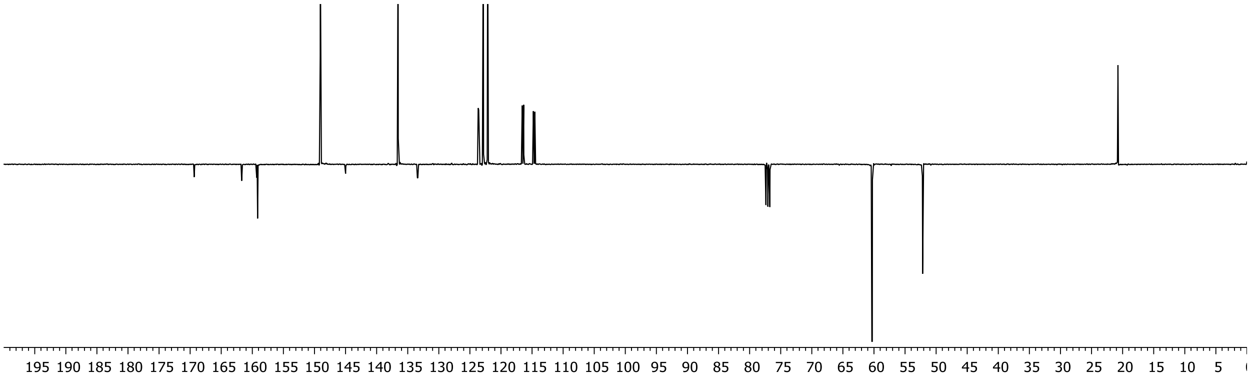

Figure S31. DEPTQ{^{1}H} NMR (100.67 MHz, $CDCl_3$, 25 °C) spectrum of compound **2-OAc**.

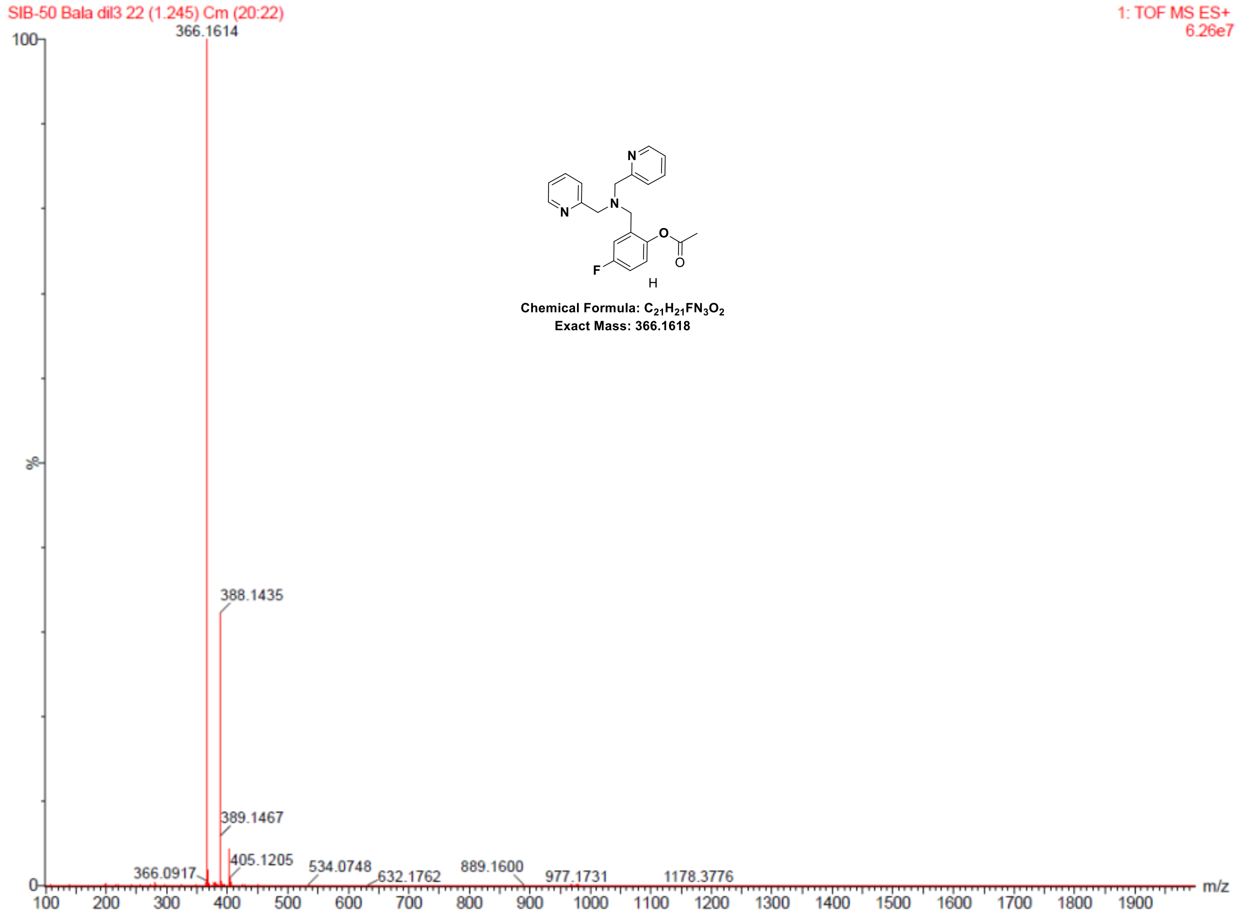

Figure S32. HRMS (ESI) spectrum of compound **2-OAc**.

G. Supplementary References.

1. Sheldrick, G. M. SHELXT -Integrated space-group and crystal structure determination. *Acta Cryst. A*. **2015**, *A64*, 112-122.
2. Sheldrick, G. M. Crystal structure refinement with SHELXL. *Acta Cryst. C.* **2015**, *C71*, 3-8.
3 O. V. Dolomanov, L. J. Bourhis, R. J. Gildea, J. A. K. Howard and H. Puschmann. OLEX2: a complete structure solution, refinement and analysis program. *J. Appl. Crystallogr*. **2009**, *42*, 339-341.
